# Publisher Correction: Eliminating blood oncogenic exosomes into the small intestine with aptamer-functionalized nanoparticles

**DOI:** 10.1038/s41467-019-13863-2

**Published:** 2020-01-08

**Authors:** Xiaodong Xie, Huifang Nie, Yu Zhou, Shu Lian, Hao Mei, Yusheng Lu, Haiyan Dong, Fengqiao Li, Tao Li, Bifei Li, Jie Wang, Min Lin, Chaihung Wang, Jingwei Shao, Yu Gao, Jianming Chen, Fangwei Xie, Lee Jia

**Affiliations:** 1 Cancer Metastasis Alert and Prevention Center, College of Chemistry; Fujian Provincial Key Laboratory of Cancer Metastasis Chemoprevention and Chemotherapy, Fuzhou University, Minjiang University Fuzhou, Fujian 350116 China; 2 Institute of Oceanography, Minjiang University, Fuzhou, Fujian 350108 China; 3 Fujian Key Laboratory for Translational Research in Cancer and Neurodegenerative Diseases, Institute for Translational Medicine, School of Basic Medical Sciences, Fujian Medical University, Fuzhou, Fujian 350108 China; 4 Department of Oncology, Fuzhou General Hospital, Fuzhou, Fujian 350001 China

**Keywords:** Lung cancer, Cancer

Correction to: *Nature Communications* 10.1038/s41467-019-13316-w, published online 02 December 2019.

The original version of this Article contained an error in the author affiliations. Affiliation 1 incorrectly read ‘Cancer Metastasis Alert and Prevention Center, College of Chemistry; Fujian Provincial Key Laboratory of Cancer Metastasis Chemoprevention and Chemotherapy, Fuzhou University, Fuzhou, Fujian 350116, China', and affiliation 2 incorrectly read ‘Institute of Oceanography, Minjiang College, Fuzhou, Fujian 350108, China’. This has now been corrected in both the PDF and HTML versions of the Article.

