# Supplementary material for: Eliminating blood oncogenic exosomes into the small intestine with aptamer-functionalized nanoparticles
Source: Nat Commun. 2019 Dec 2;10:5476. doi: 10.1038/s41467-019-13316-w (PMC6889386; doi:10.1038/s41467-019-13316-w)
Supplement: Supplementary file 1 — Supplementary Information [file 41467_2019_13316_MOESM1_ESM.pdf]

SUPPLEMENTARY INFORMATION

Eliminating blood oncogenic exosomes via the small intestine with aptamer-functionalized nanoparticles

Xiaodong Xie et al.

SUPPLEMENTARY FIGURES

Supporting Information

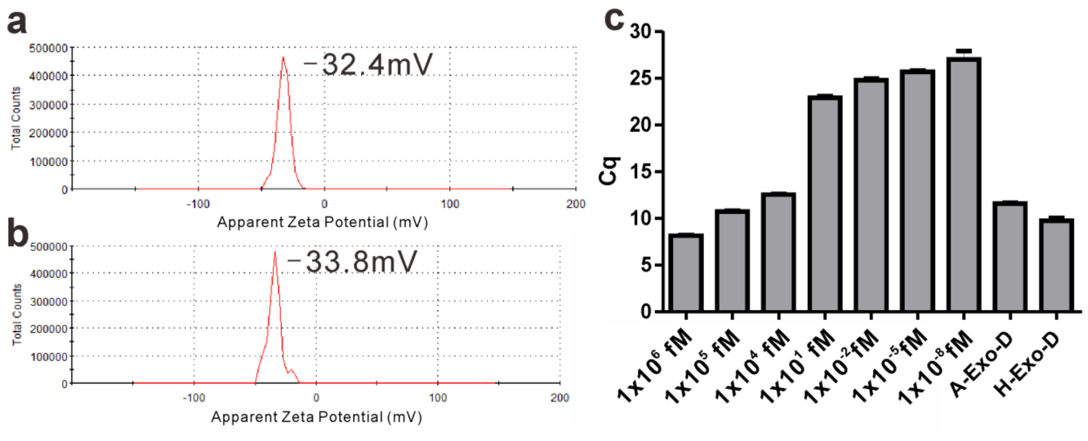

Supplementary Fig. 1. **Engineering and charactering extraneous exosomes.** a., b. Zeta potential distribution analysis of A-Exo (a) and H-Exo (b). c. qRT-PCR analysis to determine the content of foreign DNA in exosomes. Bars represent mean ± s.e.m. (n=3 each well). Source data are provided as a Source Data file.

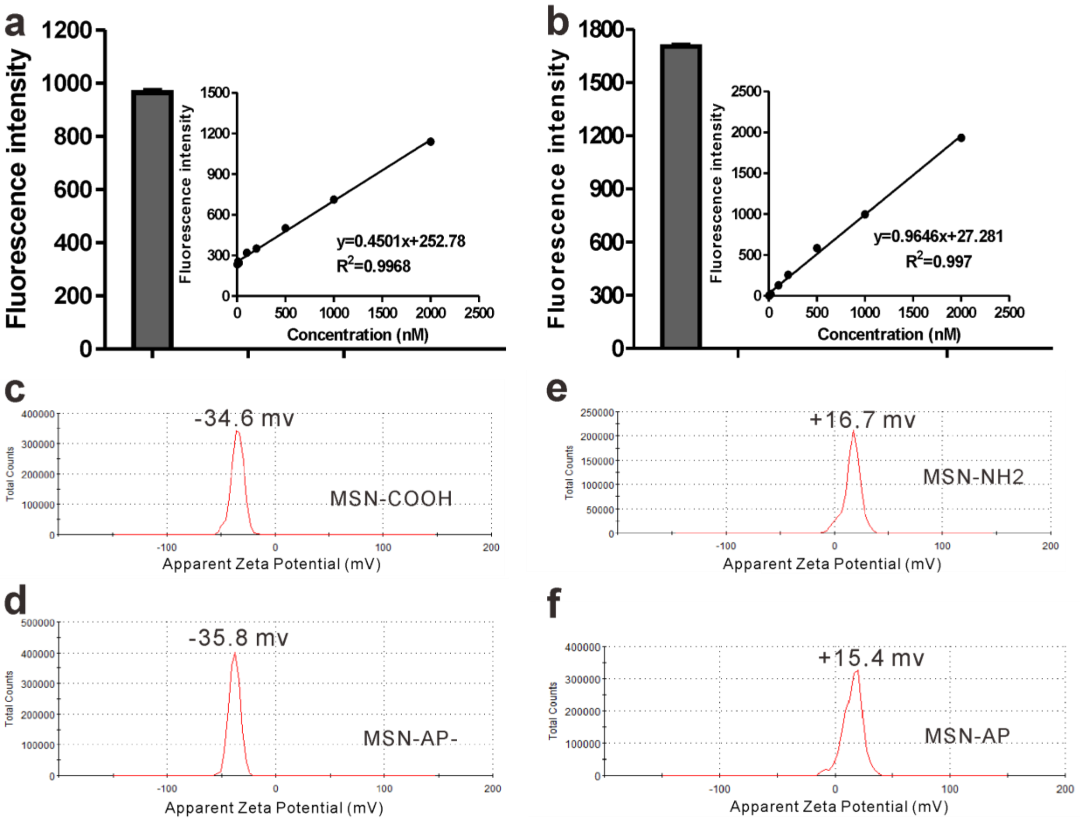

Supplementary Fig. 2. **Characterization of targeted nanoparticles.** a., b. Aptamers quantification using the standard concentration curves of Cy 3-labelled MSN-AP- (a), and Cy 5-labelled MSN-AP (b) for quantitative analysis of aptamers conjugated on the MSN. n=3. c., d. Zeta potential distribution analysis of MSN-COOH (c) and MSN-AP- (d); e., f, Zeta potential distribution analysis of MSN-NH2 (e) and MSN-AP (f). Bars represent mean ±s.e.m.(n=3). Source data are provided as a Source Data file.

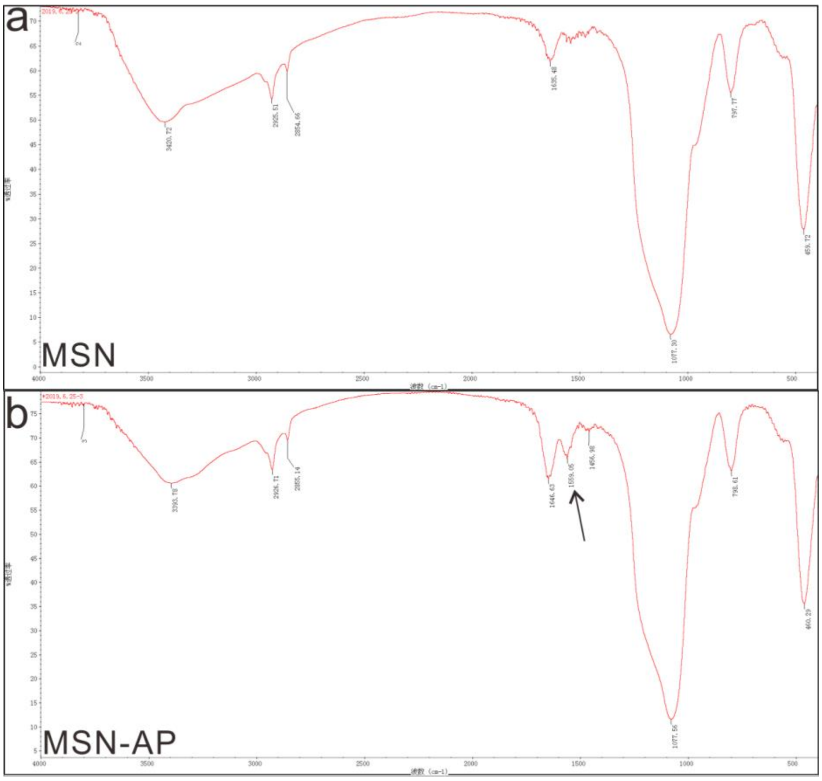

Supplementary Fig. 3. The FTIR spectra of MSN(a) and MSN-AP(b). The arrow shows AP-related spectral feature at 1559 cm^{-1} . Source data are provided as a Source Data file.

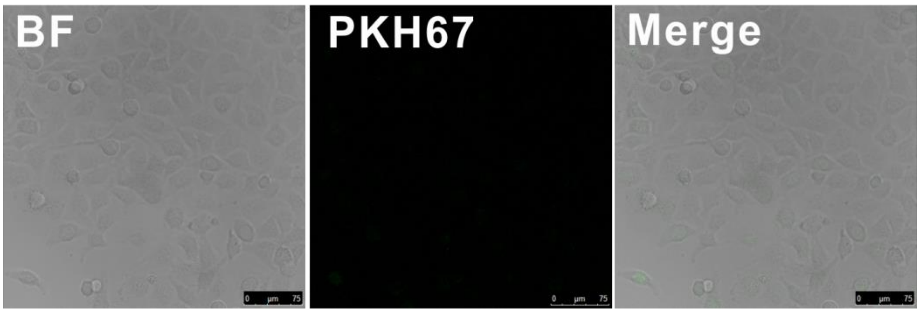

Supplementary Fig. 4. No significant binding between cationized MSN and A-Exo. Cationized MSN was incubataed with PKH67-labelled A-Exo for 1 h, and centrifuged at 15,000 g followed by examing the pellet by using a confocal dish with LO2 cells on it as a contrast backgrand. The merged results show no siginficant binding between cationized MSN and A-Exo. Source data are provided as a Source Data file.

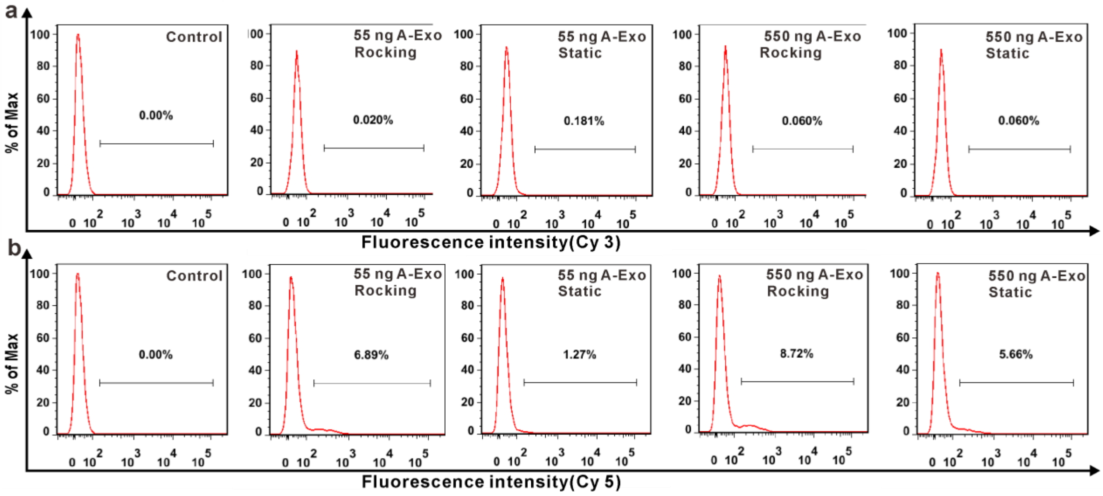

Supplementary Fig. 5. **Ability of MSN-AP and MSN-AP- to capture A-Exo in blood.** a., b. Flow cytometry quantification of A-Exo captured by MSN-AP- (a) or MSN-AP (b) in rat blood after 1-h static or rocking (100 rpm) incubation at 37˚C. MSN-AP captured more A-Exo than MSN-AP- did, and the capture was A-Exo concentration-dependent. Source data are provided as a Source Data file.

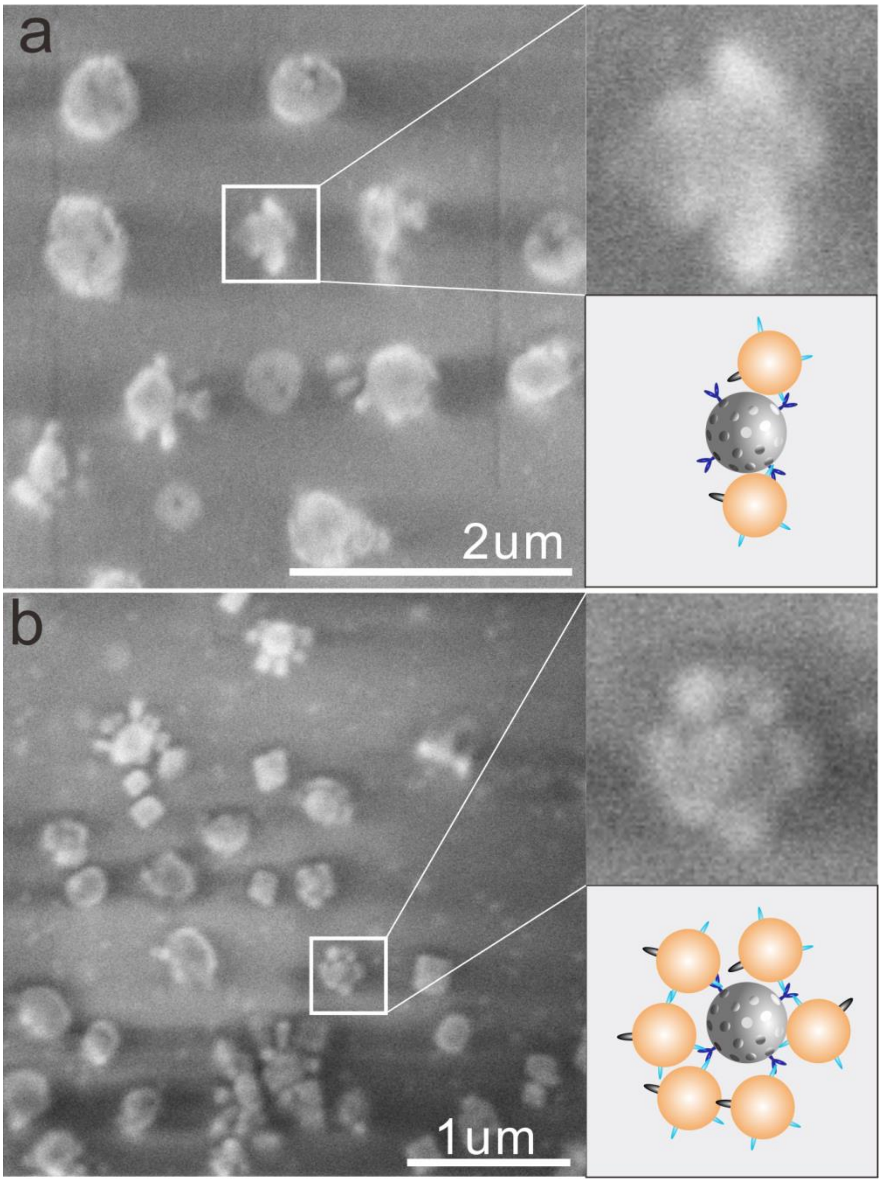

Supplementary Fig. 6. SEM images with wide-field view show conjugation between MSN-AP and A-Exo in the rat blood, which contains normal exosomes, under the static (a) and rocking (b) conditions. Source data are provided as a Source Data file.

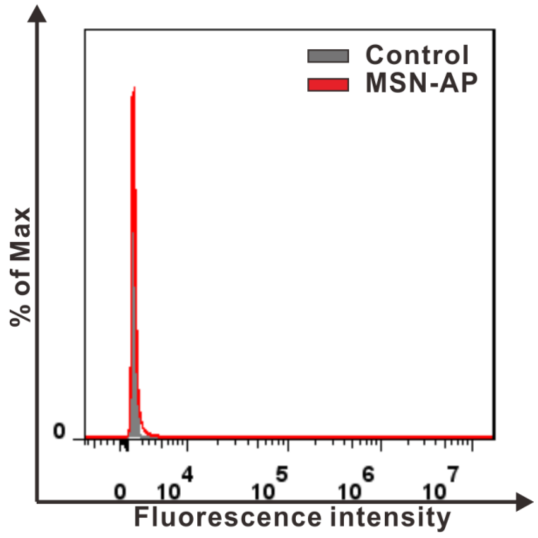

Supplementary Fig. 7. No conjugation between MSN-AP and blood exosomes. MSN-AP-Cy was incubated with 200 µl of rat blood, and mixed at 100 rpm for 4 h at 37 ℃. The suspension was diluted to 1 ml with PBS and centrifuged at 200 g for 5 min. The supernatant was centrifuged at 15,000 g for 10 min to collect the deposit that was incubated with 10 µl of anti-CD9 beads and washed twice with PBS, and suspended in 500 µl of PBS. The flow cytometry results show no conjugate between MSN-AP and blood normal exosomes. Source data are provided as a Source Data file.

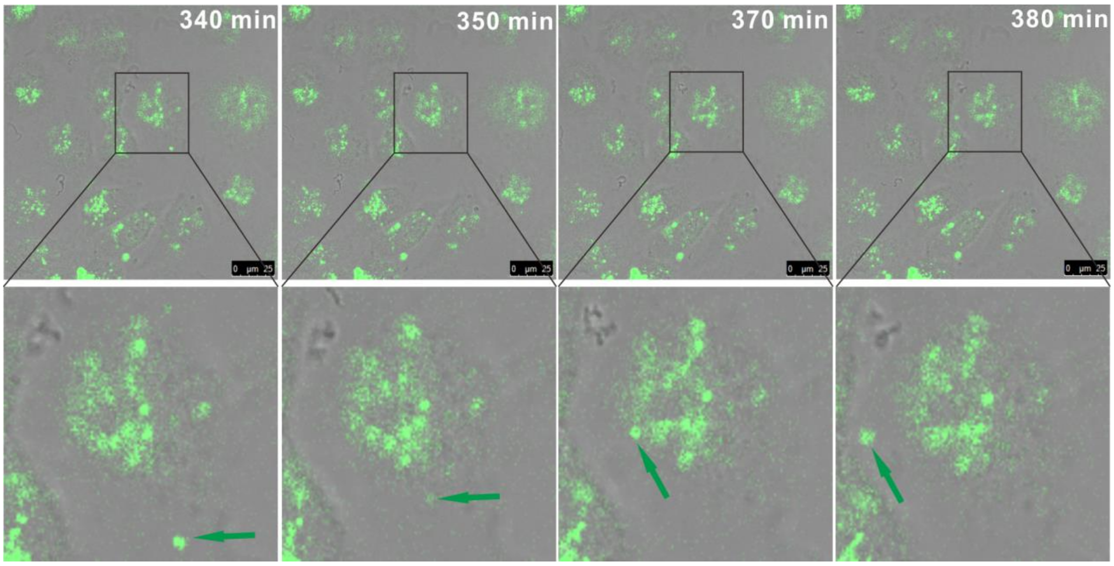

Supplementary Fig. 8. The time-lapse image sequences of confocal microscope show the uptake and exocytosis of PKH67-A-Exo alone by LO2 cells (green arrows). Source data are provided as a Source Data file.

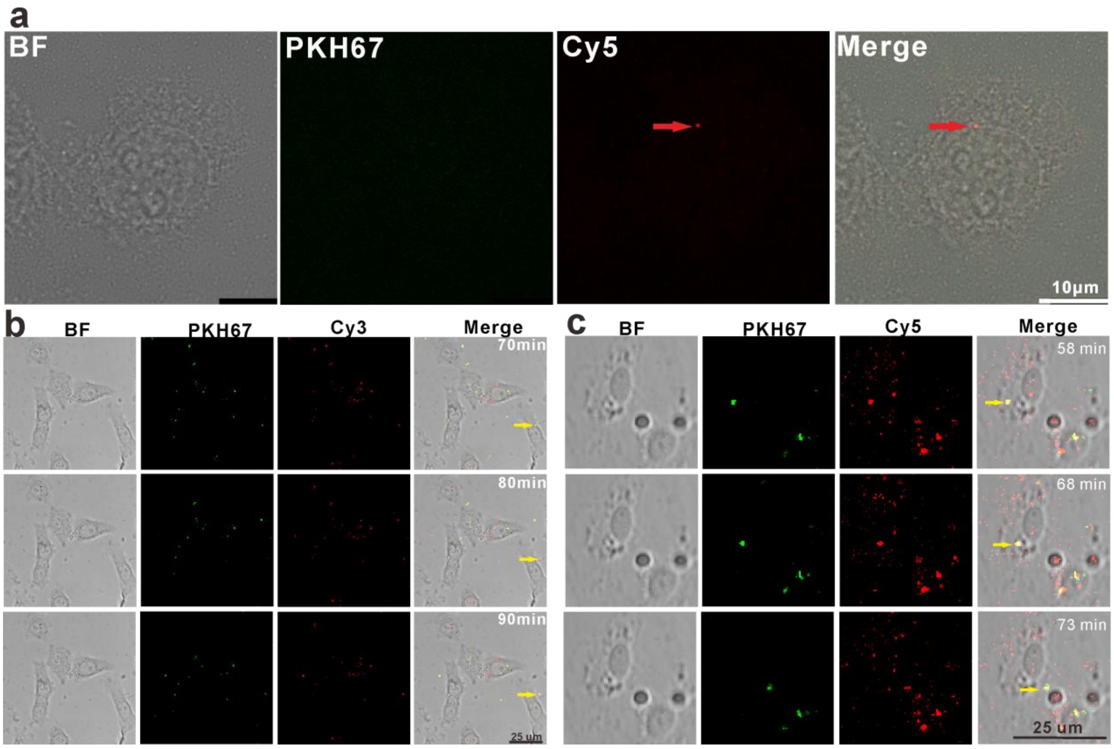

Supplementary Fig. 9. In vitro binding between MSN-AP and A-Exo and dynamic trafficking of MSN-Exo through LO2 cells. a. Confocal microscopy images of Cy-5-labelled MSN-AP incubated with PKH67-labelled A-Exo. The resulted products (MSN-Exo, MSN-AP) were examined for their endocytosis by hepatocytes LO2 under the confocal microscope. Red dots represent MSN-AP. b., c. Trafficking of the MSN-Exo within the same LO2 cell. MSN-Exo formed after 1-h incubation of MSN-AP- (b) or MSN-AP (c) with A-Exo. The time lapse image sequences of confocal microscopy show the dynamic trafficking of the MSN-Exo (arrows indicate) in and out the same LO2 cell at different minutes. Note, the red dots represent MSN-AP or MSN-AP-. The green dots represent A-Exo. Source data are provided as a Source Data file.

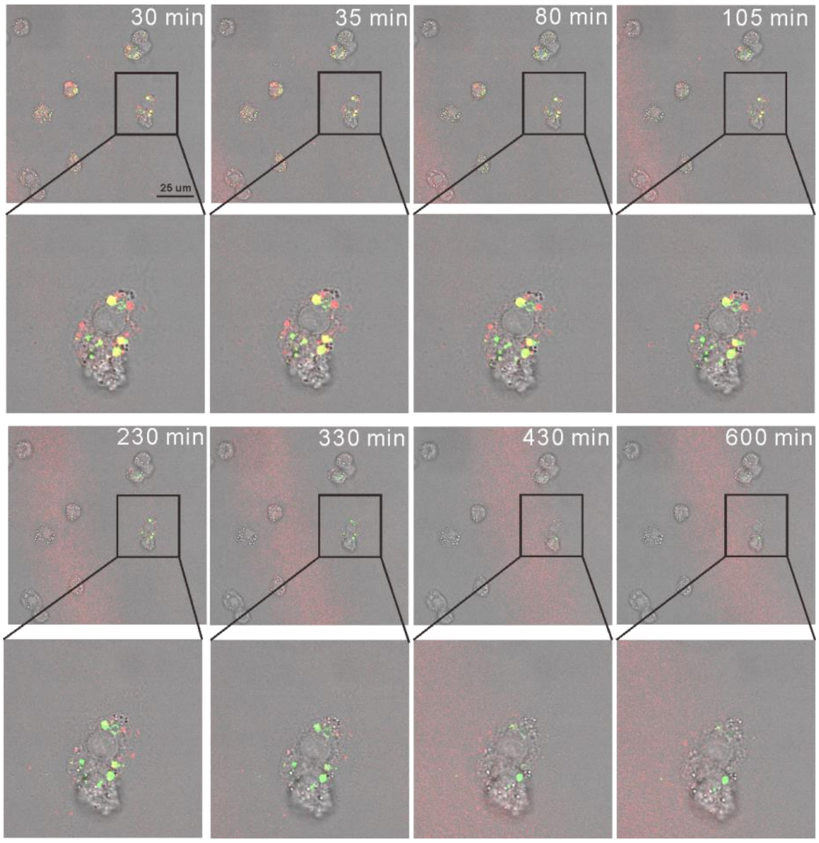

Supplementary Fig. 10. Digestion dynamics of the formed MSN-Exo by a Kupffer cell. The time lapse image sequences of confocal microscopy show the digestion by a Kupffer cell of the MSN-Exo- formed by the conjugation between MSN-AP- and A-Exo within 1-h incubation. The photos were taken at different time points. The lower panels represent the amplification of the upper ones. Source data are provided as a Source Data file.

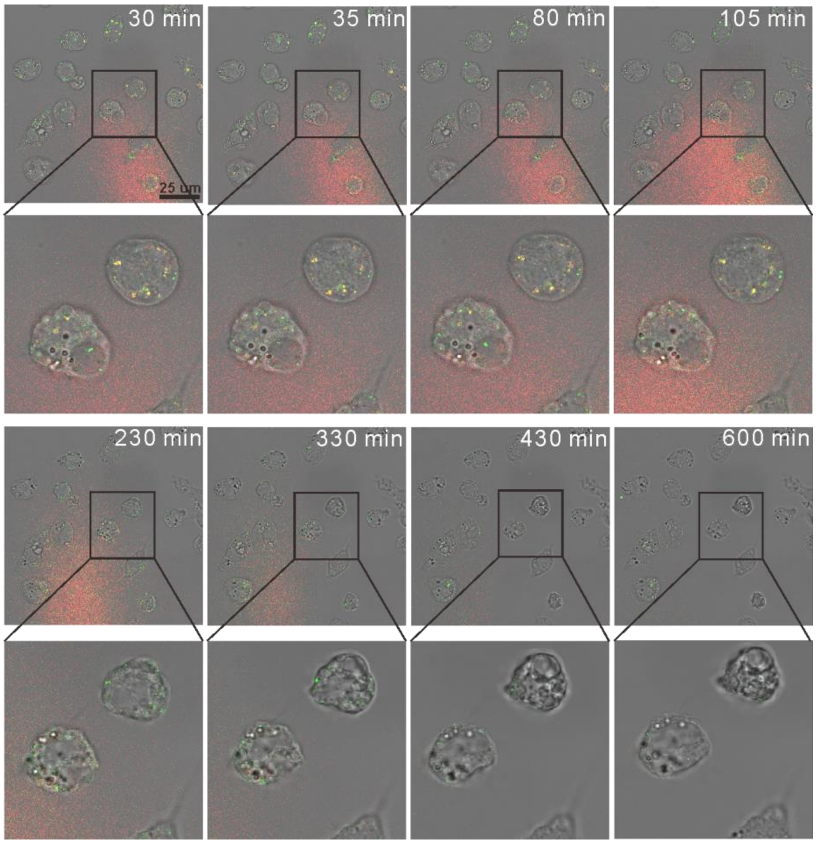

Supplementary Fig. 11. Digestion dynamics of the formed MSN-Exo by Kupffer cells. The time lapse image sequences of confocal microscopy show the digestion by Kupffer cells of the MSN-Exo formed by the conjugation between the positive Cy5-labelled MSN-AP and PKH67-labelled A-Exo within 1-h incubation. The photos were taken at different time points. The lower panels represent the amplification of the upper ones. Source data are provided as a Source Data file.

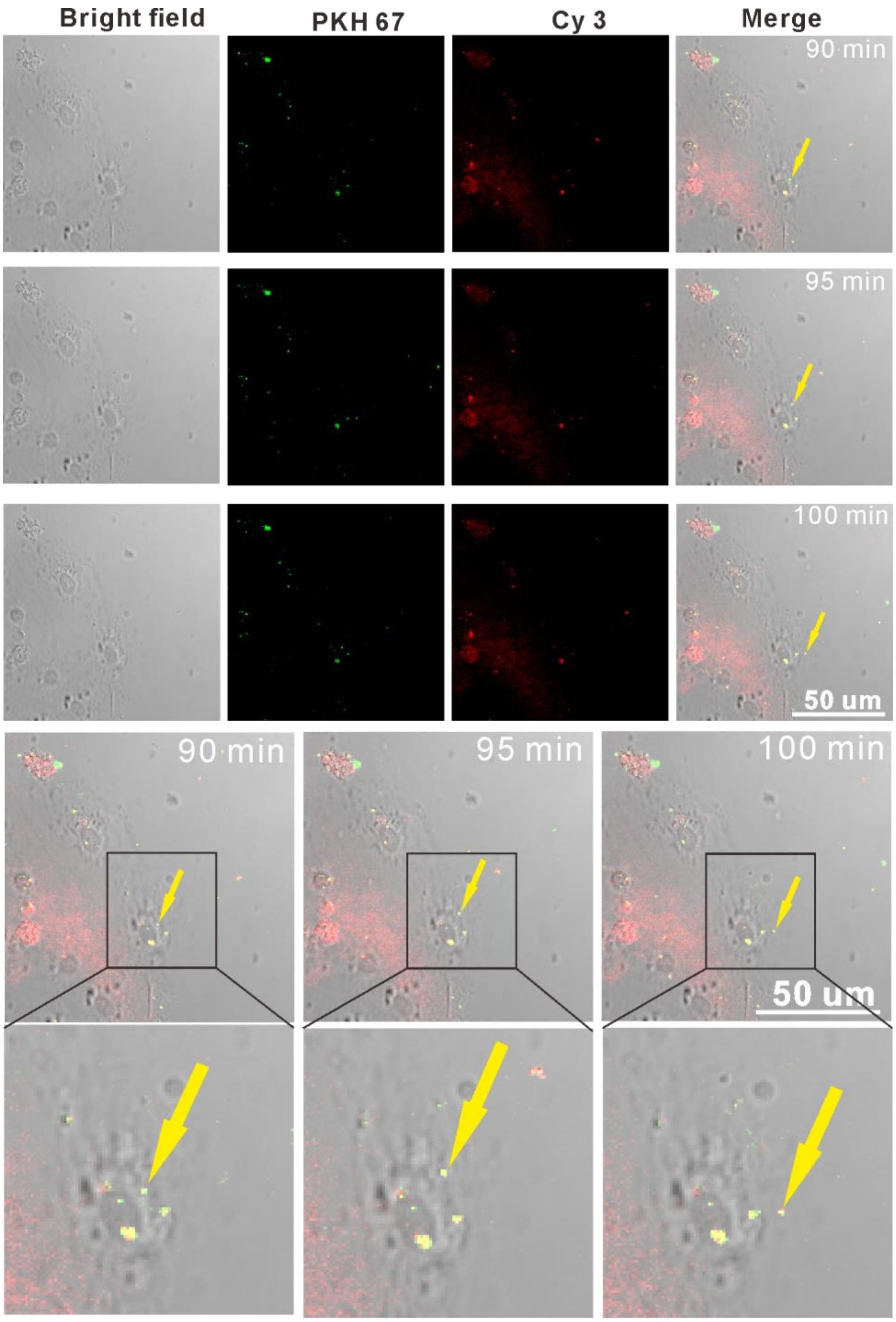

Supplementary Fig. 12. Trafficking of the negatively-charged MSN-Exo- in and out of the same endothelial cell. Time lapse image sequences of the merged confocal microscopy (far left) show the trafficking of the same MSN-Exo- within (90 min), at the edge of (95 min), and out (100 min) the same endothelial cell. The lower two panels represent the amplification of the left merged upper ones. The MSN-Exo- were formed after 1-h incubation of Cy3-labelled MSN-AP- with PKH67-labelled A-Exo. Source data are provided as a Source Data file.

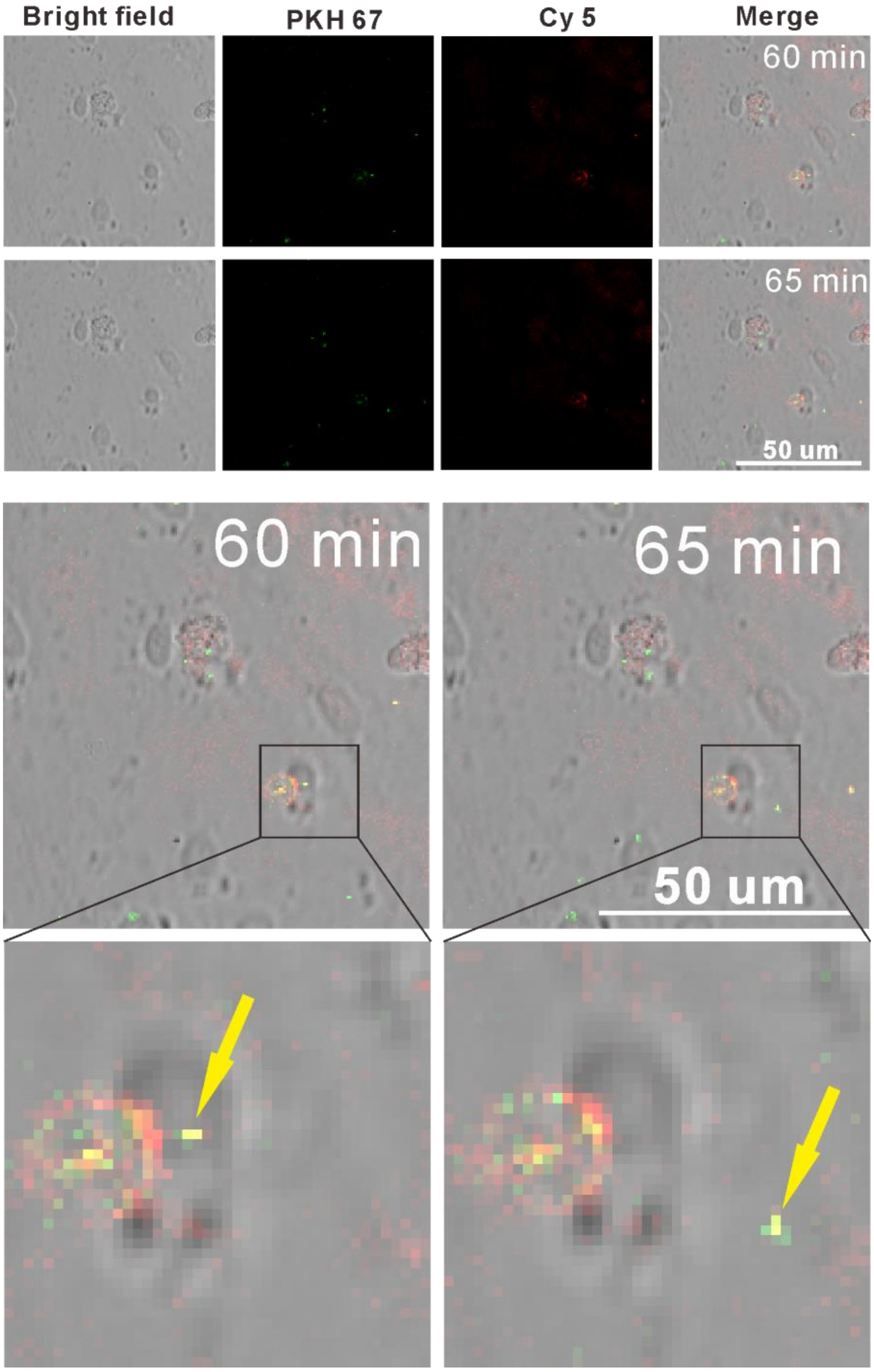

Supplementary Fig. 13. Trafficking of the positively-charged MSN-Exo in and out of the same endothelial cell. Time lapse image sequences of the merged confocal microscopy (far left) show the trafficking of the same MSN-Exo- within (60 min) and out the same endothelial cell. The lower two panels represent the amplification of the left merged upper ones. The MSN-Exo were formed after 1-h incubation of Cy5-labelled MSN-AP with PKH67-labelled A-Exo. Source data are provided as a Source Data file.

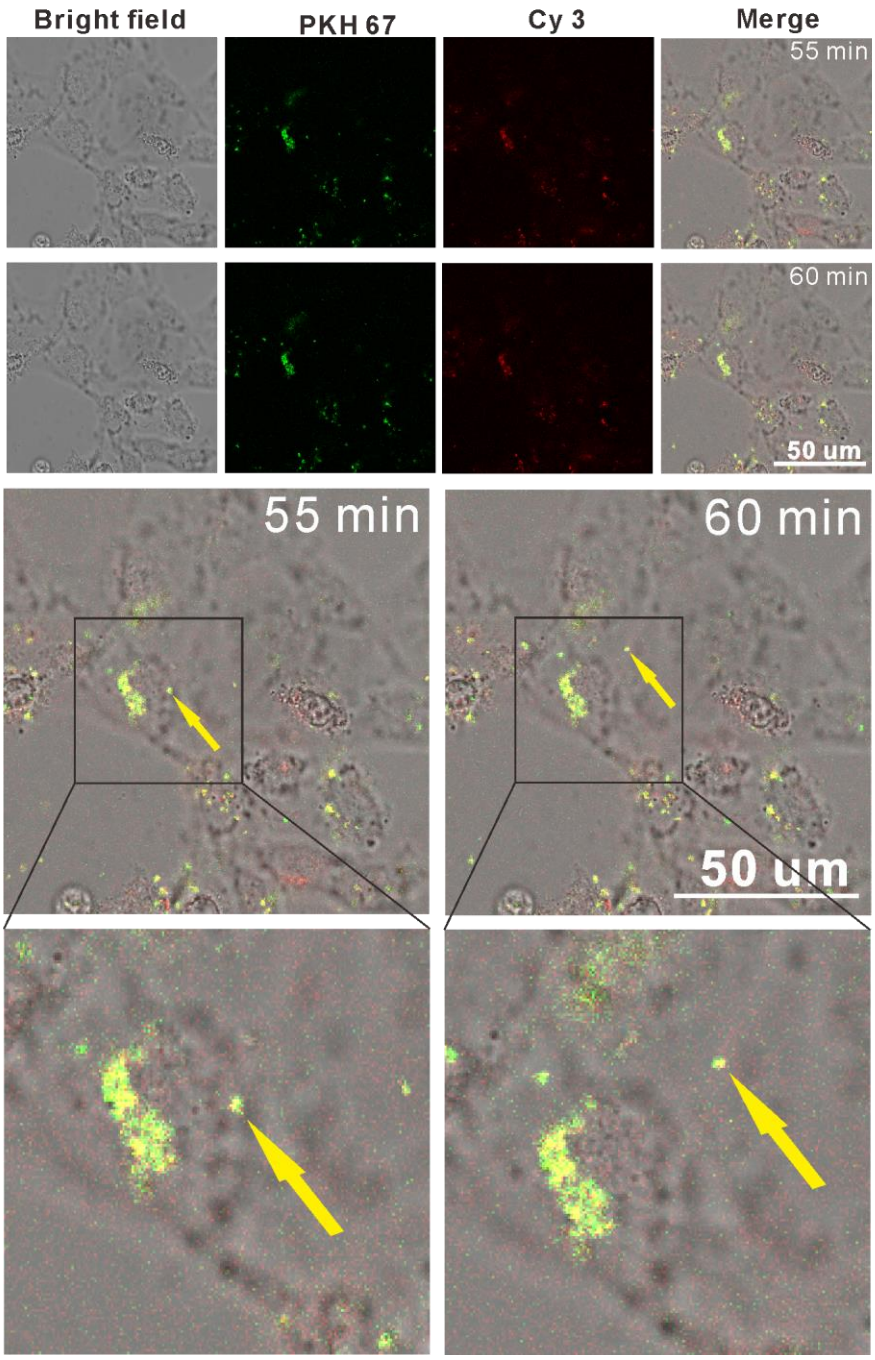

Supplementary Fig. 14. Trafficking of the negatively-charged MSN-Exo- in and out of the same cholangiocyte. Time lapse image sequences of the merged confocal microscopy (far left) show the trafficking of the same MSN-Exo- within (55 min) and out (60 min) the same cholangiocyte. The lower two panels represent the amplification of the left merged upper ones. The MSN-Exo- were formed after 1-h incubation of Cy3-labelled MSN-AP- with PKH67-labelled A-Exo. Source data are provided as a Source Data file.

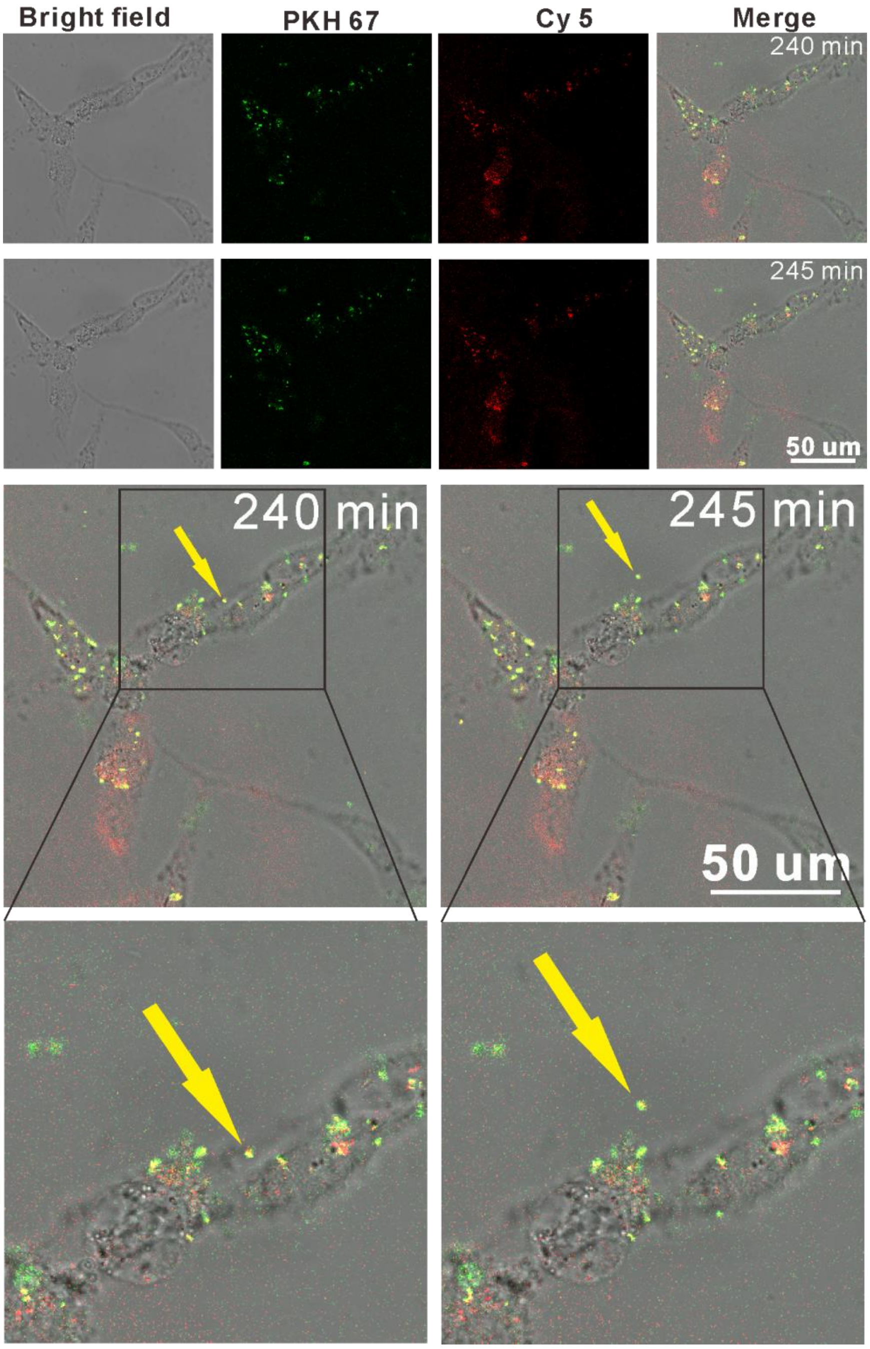

Supplementary Fig. 15. Trafficking of the positively-charged MSN-Exo in and out of the same cholangiocyte. Time lapse image sequences of the merged confocal microscopy (far left) show the trafficking of the same MSN-Exo within (240 min) and out (245 min) the same cholangiocyte. The lower two panels represent the amplification of the left merged upper ones. The MSN-Exo were formed after 1-h incubation of Cy5-labelled MSN-AP with PKH67-labelled A-Exo. Source data are provided as a Source Data file.

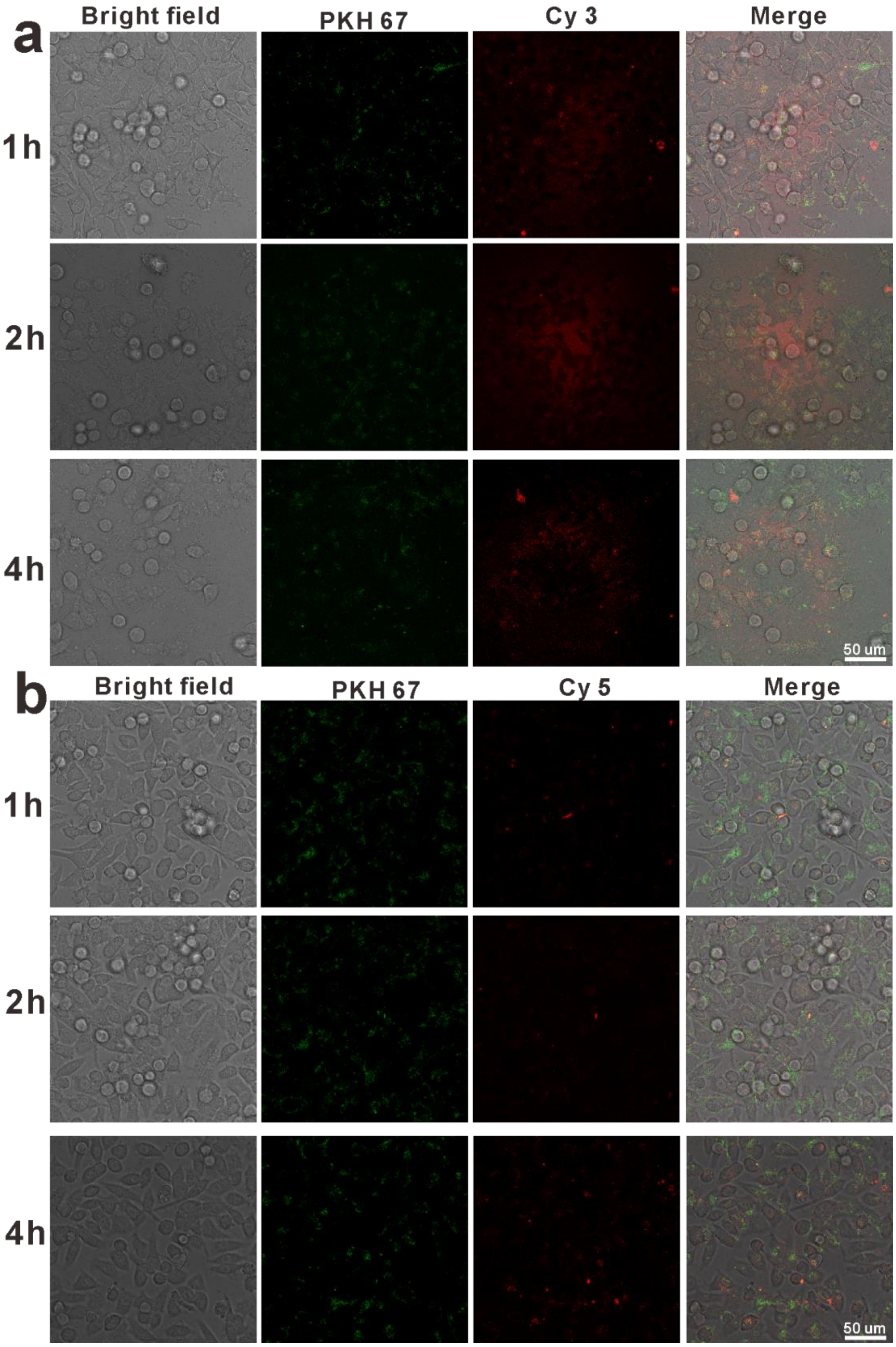

Supplementary Fig. 16. Transcellular permeability of MSN-Exo across Kupffer and LO2 monolayers. a., b. Confocal microscopy images show the formation of cell monolayers after co-incubation of Kupffer with LO2 (cell ratio= 1:6) on the upper chamber of transwell. Cy3-labelled MSN-AP- (a), or Cy5- labelled MSN-AP (b) were pre-incubated with A-Exo, the products were added on to the upper chamber. After 1, 2 and 4 h, 200 μl of the medium samples were collected from the lower chamber and added into a new confocal dish with LO2 cells on it for quantification of the permeated MSN-Exo. Source data are provided as a Source Data file.

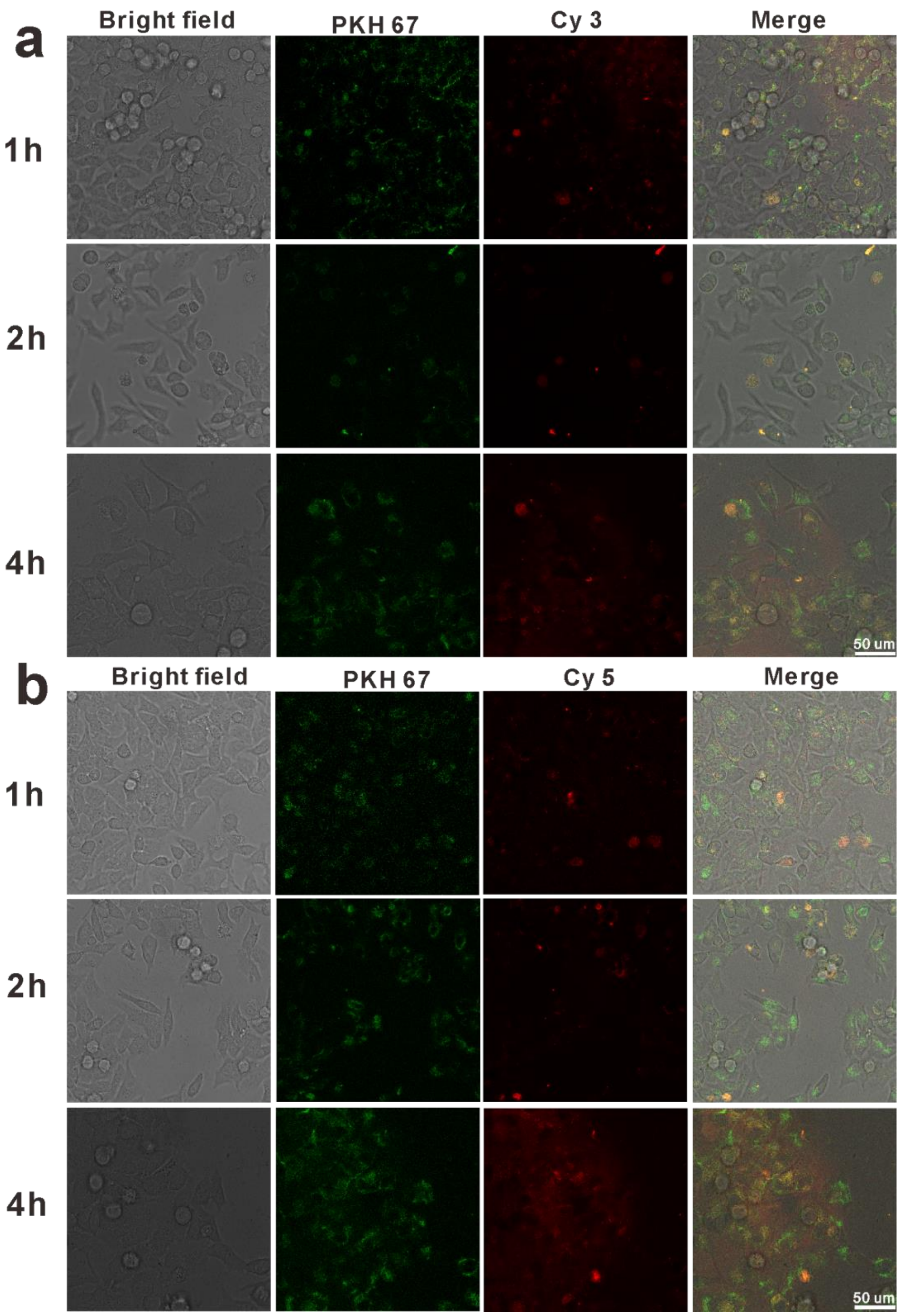

Supplementary Fig. 17. Transcellular permeability of MSN-Exo across Kupffer and endothelial monolayers. a., b. Confocal microscopy images show the formation of cell monolayers after co-incubation of Kupffer with endothelial cells (cell ratio= 1:6) on the upper chamber of transwell. Cy3-labelled MSN-AP- (a), or Cy5- labelled MSN-AP (b) were pre-incubated with A-Exo, the products were added on to the upper chamber. After 1, 2 and 4 h, 200 μl of the medium samples were collected from the lower chamber and added into a new confocal dish with LO2 cells on it for quantification of the permeated MSN-Exo. Source data are provided as a Source Data file.

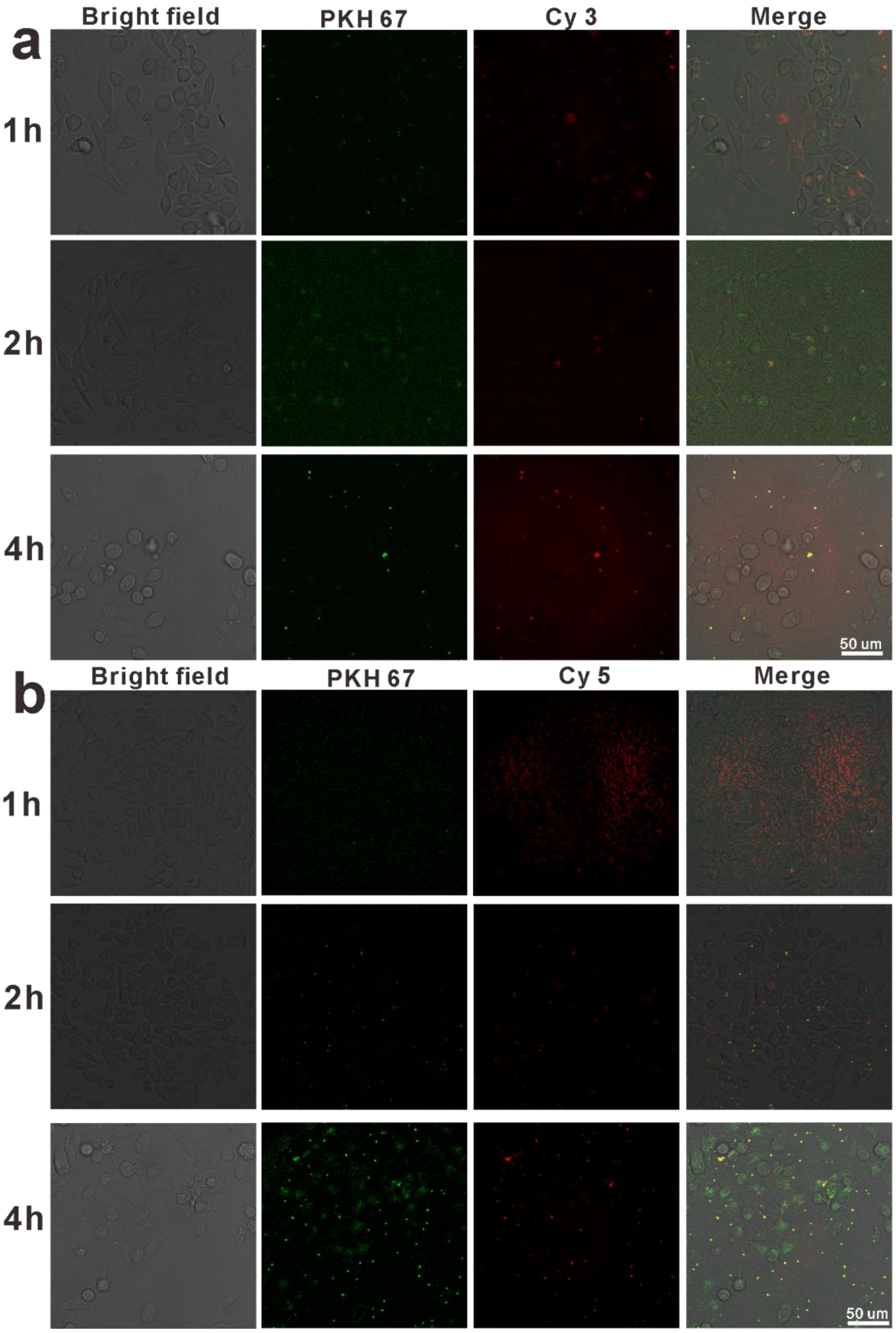

Supplementary Fig. 18. Transcellular permeability of MSN-Exo across LO2 monolayers. a., b. Confocal microscopy images show the formation of LO2 monolayers after incubated on the upper chamber of transwell. Cy3-labelled MSN-AP- (a), or Cy5-labelled MSN-AP (b) were pre-incubated with A-Exo, the products were added on to the upper chamber. After 1, 2 and 4 h, 200 μl of the medium samples were collected from the lower chamber and added into a new confocal dish with LO2 cells on it for quantification of the permeated MSN-Exo. Source data are provided as a Source Data file.

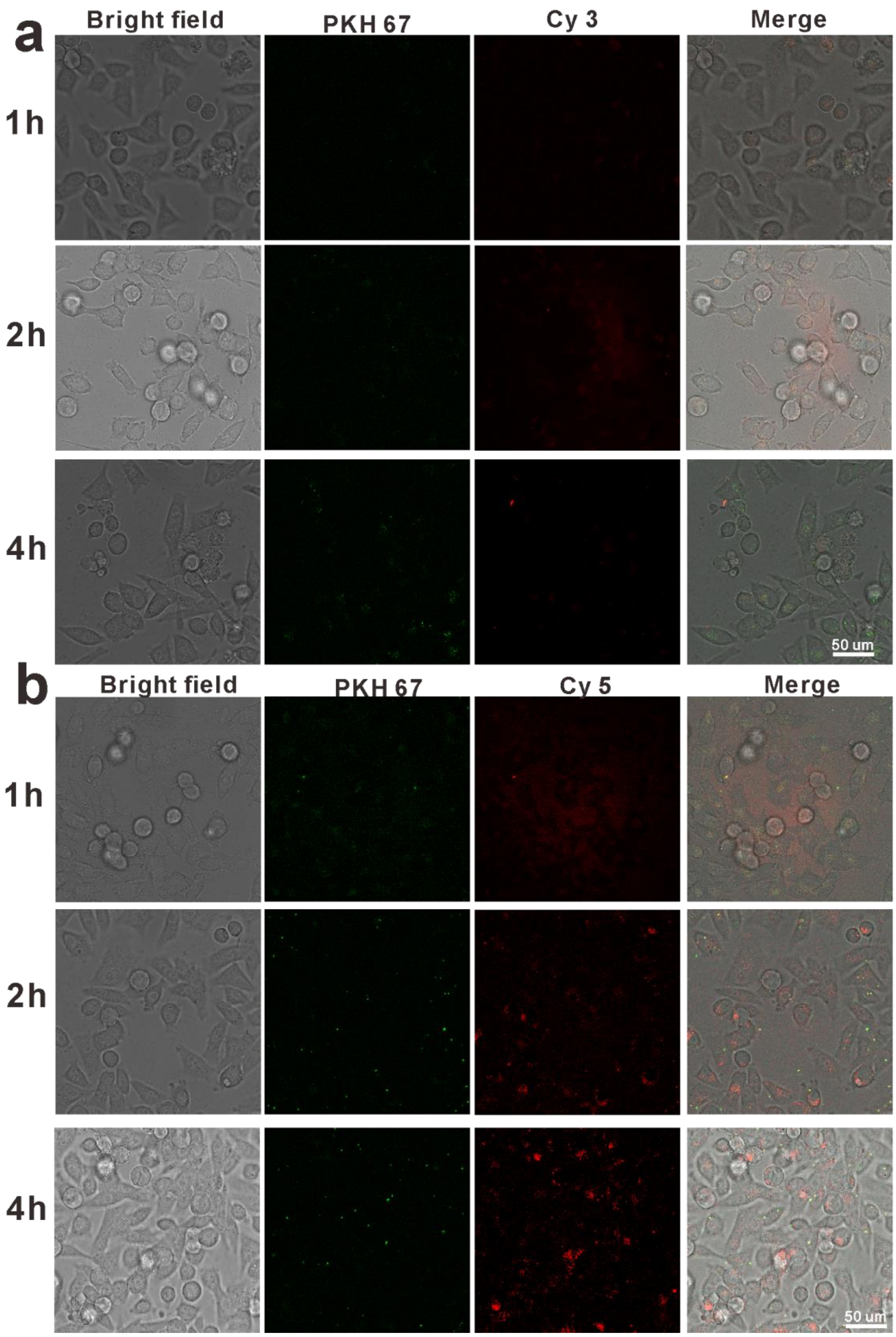

Supplementary Fig. 19. Transcellular permeability of MSN-Exo across cholangiocyte monolayers. a., b. Confocal microscopy images show the formation of cholangiocyte monolayers after incubated on the upper chamber of transwell. Cy3-labelled MSN-AP- (a), or Cy5- labelled MSN-AP (b) were pre-incubated with A-Exo, the products were added on to the upper chamber. After 1, 2 and 4 h, 200 µl of the medium samples were collected from the lower chamber and added into a new confocal dish with LO2 cells on it for quantification of the permeated MSN-Exo. Source data are provided as a Source Data file.

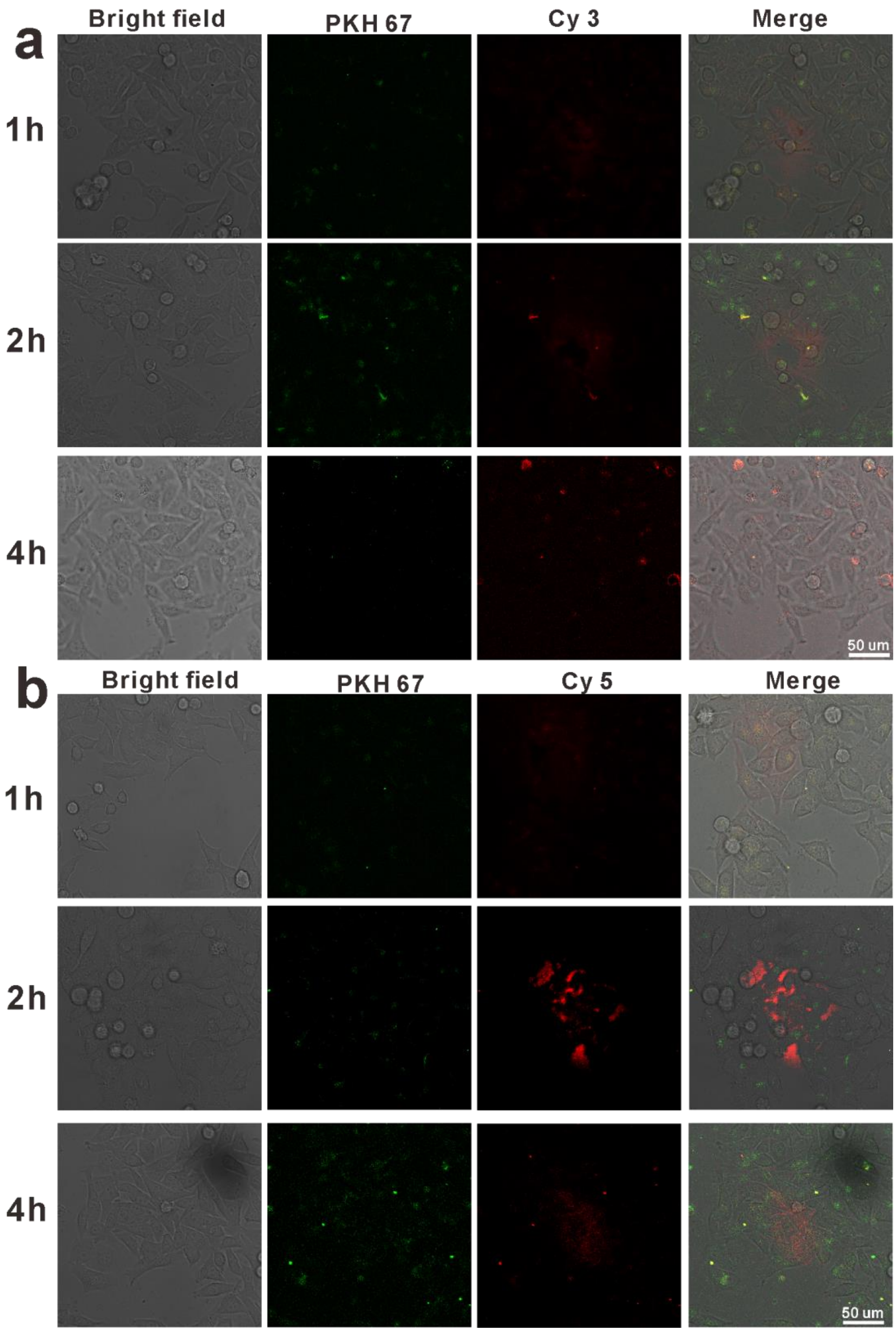

Supplementary Fig. 20. Transcellular permeability of MSN-Exo across endothelial monolayers. a., b. Confocal microscopy images show the formation of endothelial monolayers after incubated on the upper chamber of transwell. Cy3-labelled MSN-AP-(a), or Cy5- labelled MSN-AP (b) were pre-incubated with A-Exo, the products were added on to the upper chamber. After 1, 2 and 4 h, 200 μl of the medium samples were collected from the lower chamber and added into a new confocal dish with LO2 cells on it for quantification of the permeated MSN-Exo. Source data are provided as a Source Data file.

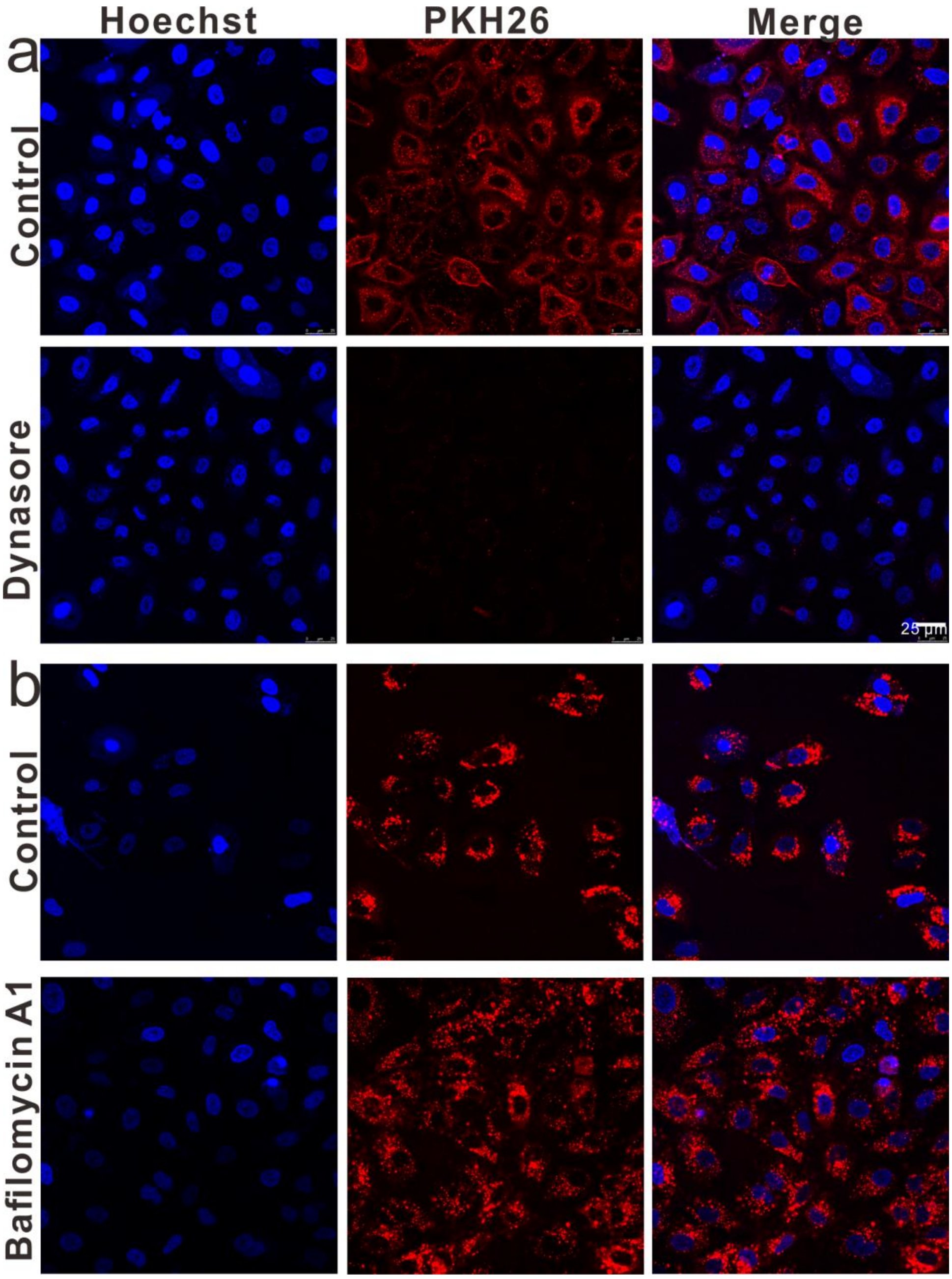

Supplementary Fig. 21. Confocal microscopy analysis of MSN-Exo transcytosis across LO2 cells. Intracellular PKH26-labelled MSN-Exo was reduced by endocytosis inhibitor Dynasore 50 μM (a), or increased by exocytosis inhibitor Bafilomycin A1 100 nM (b) in comparison with the PBS control. Source data are provided as a Source Data file.

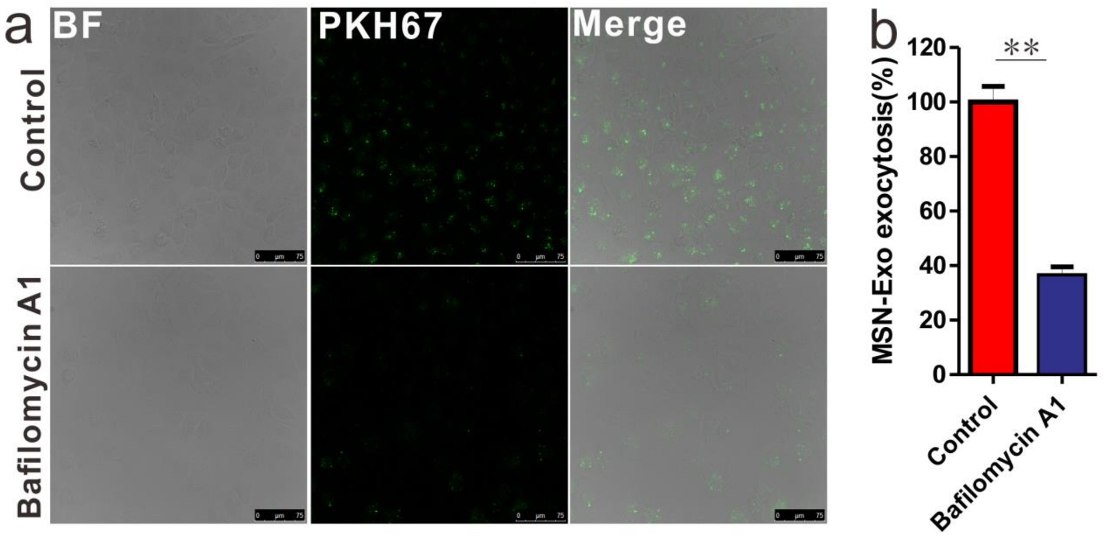

Supplementary Fig. 22. Bafilomycin A1 treatment decreases exocytosis of MSN-Exo from LO2 cells. The number of MSN-Exo that passed through the LO2 cells was evaluated by incubating LO2 cells with PKH67-labelled MSN-Exo for 30 min followed by washing the cells with PBS to discard the free PKH67-labelled MSN-Exo. The LO2 cells were treated with Bafilomycin A1 for 6 h. The number of exocytosed PKH67-labelled MSN-Exo in the culture medium was quantified by confocal microscopy in comparison with the control. Bars represent mean ± s.e.m. n=9, **, $P < 0.01$; unpaired two-tailed *t*-test. Source data are provided as a Source Data file.

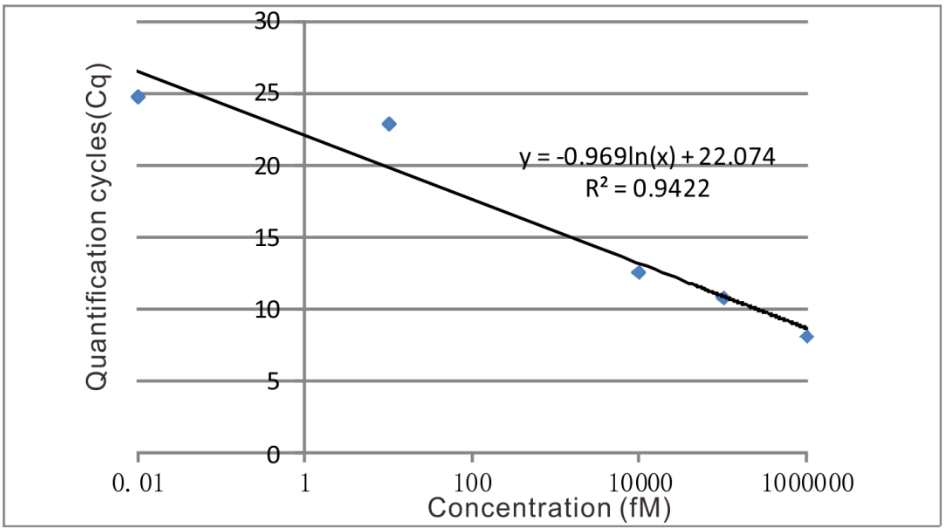

Supplementary Fig. 23. The standard curve shows relationship between amount of foreign DNA and Cq analyzed by qRT-PCR. Source data are provided as a Source Data file.

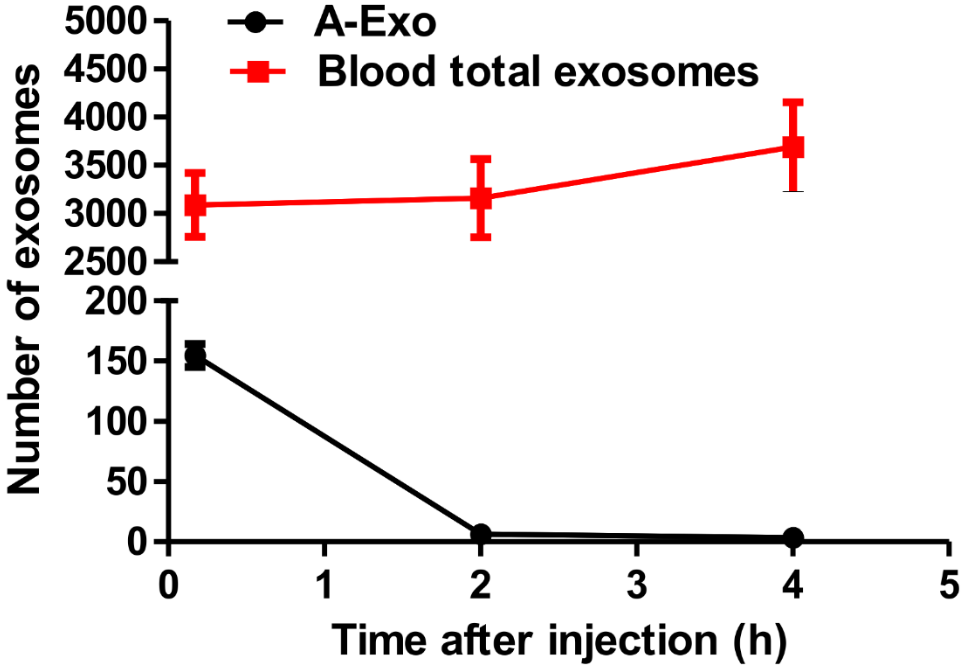

Supplementary Fig. 24. Time-course of changes of exogenous A-Exo and total normal exosomes in the tumor-bearing mouse blood after intravenous administration of MSN-AP (5 mg/kg). The Y-axis represents the average number of the 5-10 randomly–selected single fields of visions. Bars represent mean ± s.e.m. n=5 for A-Exo ; n=10 for blood total exosomes. Source data are provided as a Source Data file.

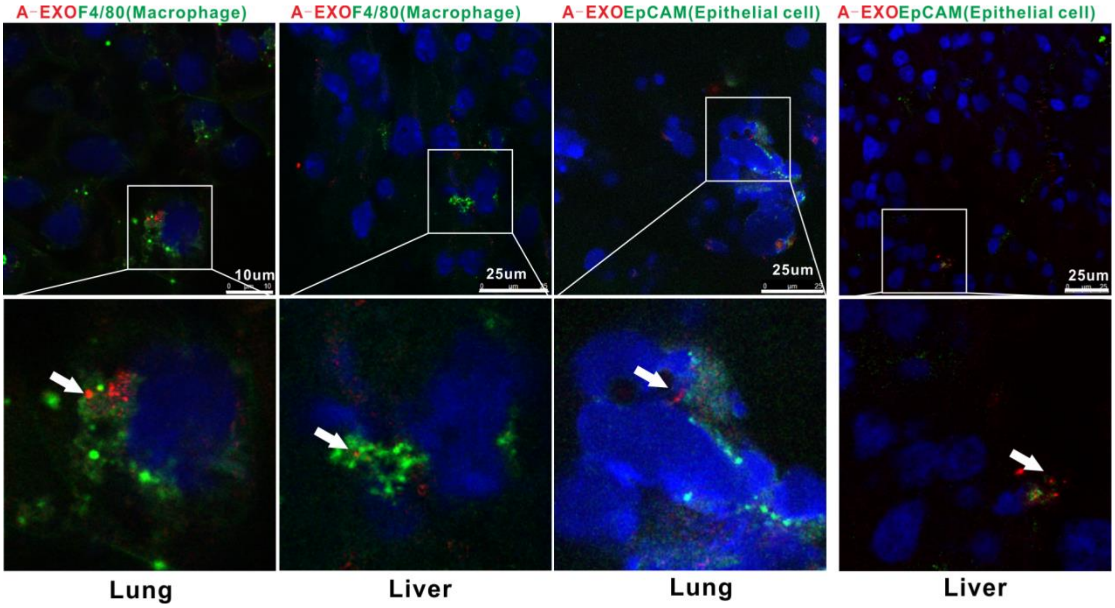

Supplementary Fig. 25. Immunofluorescent analysis of A-Exo distribution in the mouse lung and liver, where different types of cells endocytose the exosomes. Lung and liver were co-stained with PKH26-labelled exosomes (red) and anti-F4/80 (green; for macrophage) or anti-EpCAM (green; for epithelial cells). The lower panel is the magnification of the upper one. Scale bar, 25 μm. Source data are provided as a Source Data file.

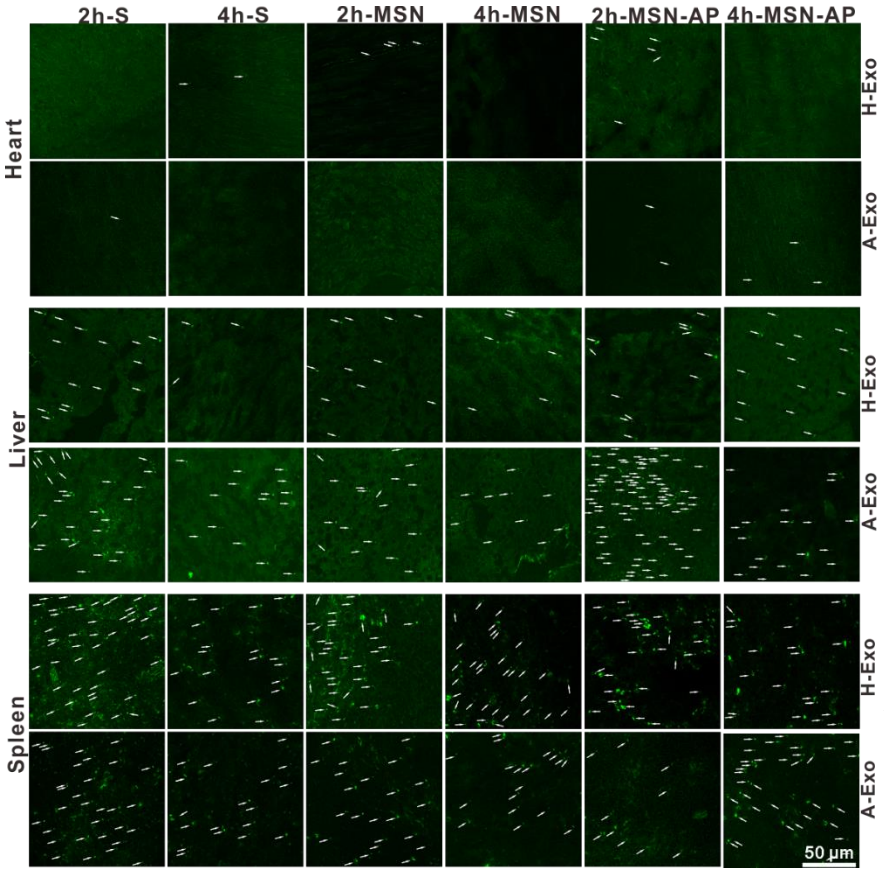

Supplementary Fig. 26. Distribution of exosomes in heart, liver and spleen. PKH67-labelled A-Exo and H-Exo (10 μg) were injected into mice through tail vein. Ten min later, saline (S; 200 μl), MSN and MSN-AP (both 5 mg/kg) were injected. Mice were sacrificed after 2 or 4 hours and then heart, liver and spleen were frozen-sectioned, and the tissue distribution of exosomes was analyzed by confocal microscopy. Source data are provided as a Source Data file.

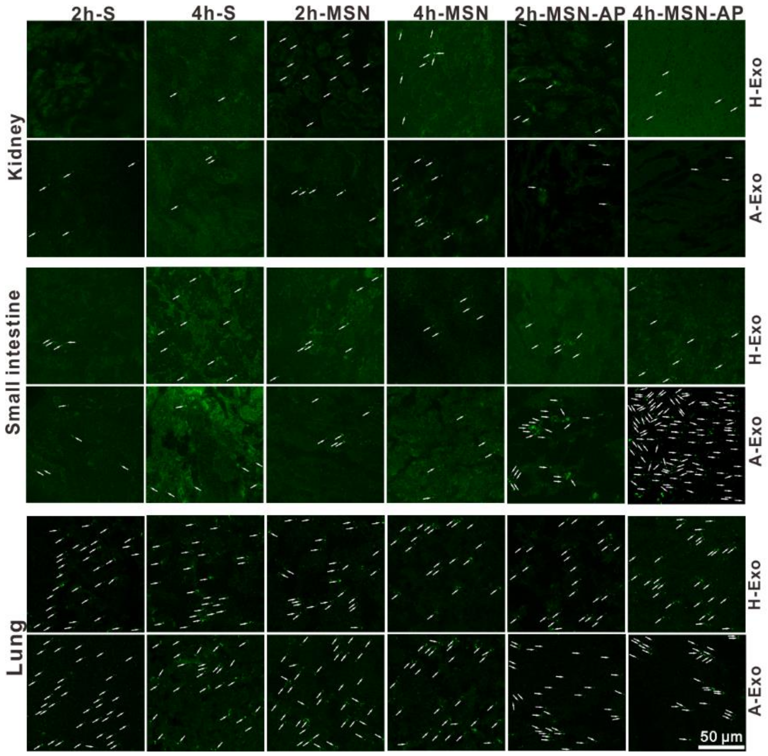

Supplementary Fig. 27. Distribution of exosomes in kidney, small intestine, lung. PKH67-labelled A-Exo and H-Exo (10 μg) were injected into mice through tail vein. Ten min later, saline (S; 200 μl), MSN and MSN-AP (both 5 mg/kg) were injected. Mice were sacrificed after 2 or 4 hours and then kidney, small intestine and lung were frozen-sectioned, and the tissue distribution of exosomes was analyzed by confocal microscopy. Source data are provided as a Source Data file.

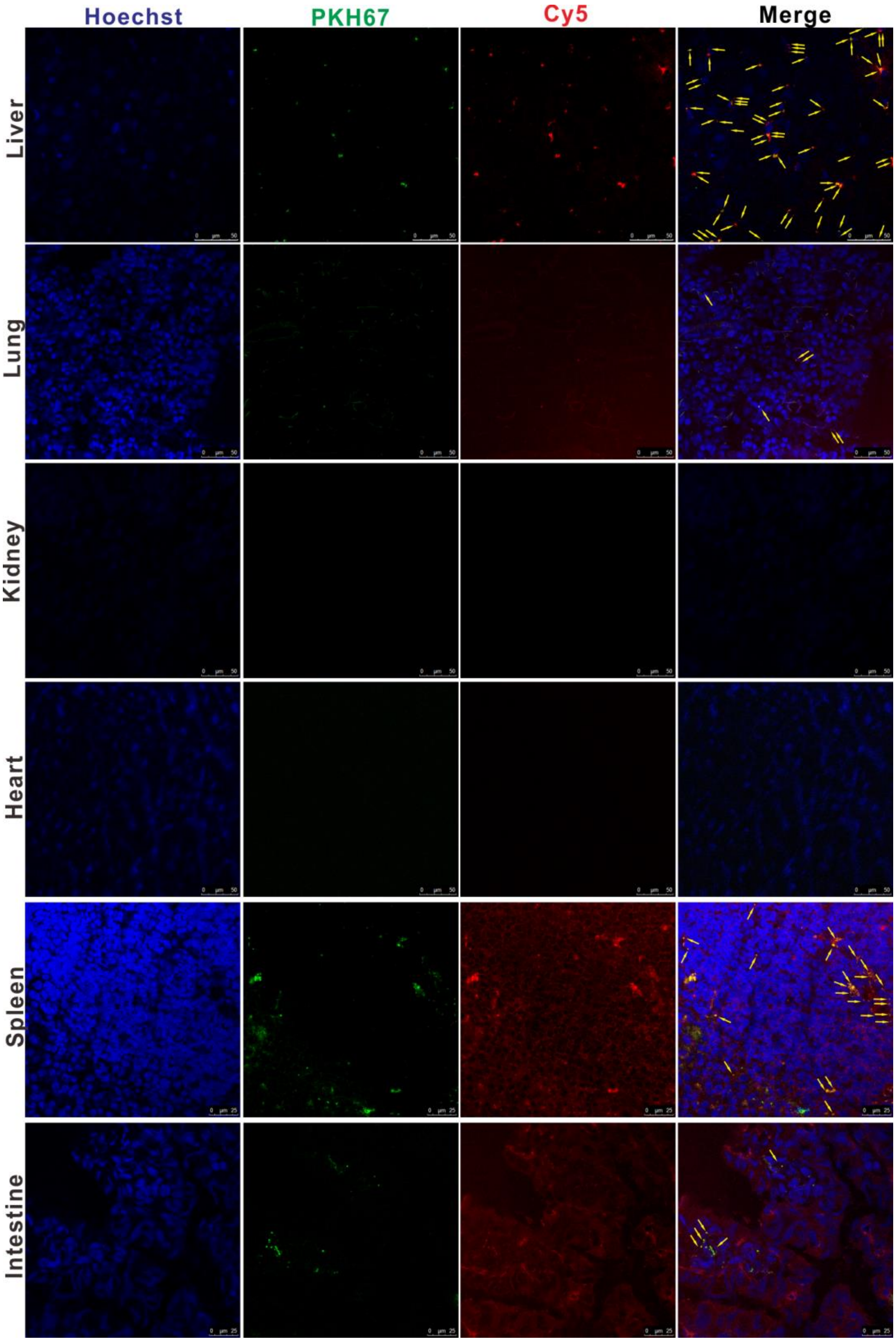

Supplementary Fig. 28. Distribution of MSN-Exo in different organs after 2 h injections of A-Exo and MSN-AP. PKH67-labelled A-Exo (10 μg) were injected into mice through tail vein. Ten min later, MSN-AP-Cy (5 mg/kg) were injected. Mice were sacrificed after 2 hours and then kidney, small intestine and lung were frozen-sectioned, and the tissue distribution of MSN-Exo (yellow) was analyzed by confocal microscopy. Source data are provided as a Source Data file.

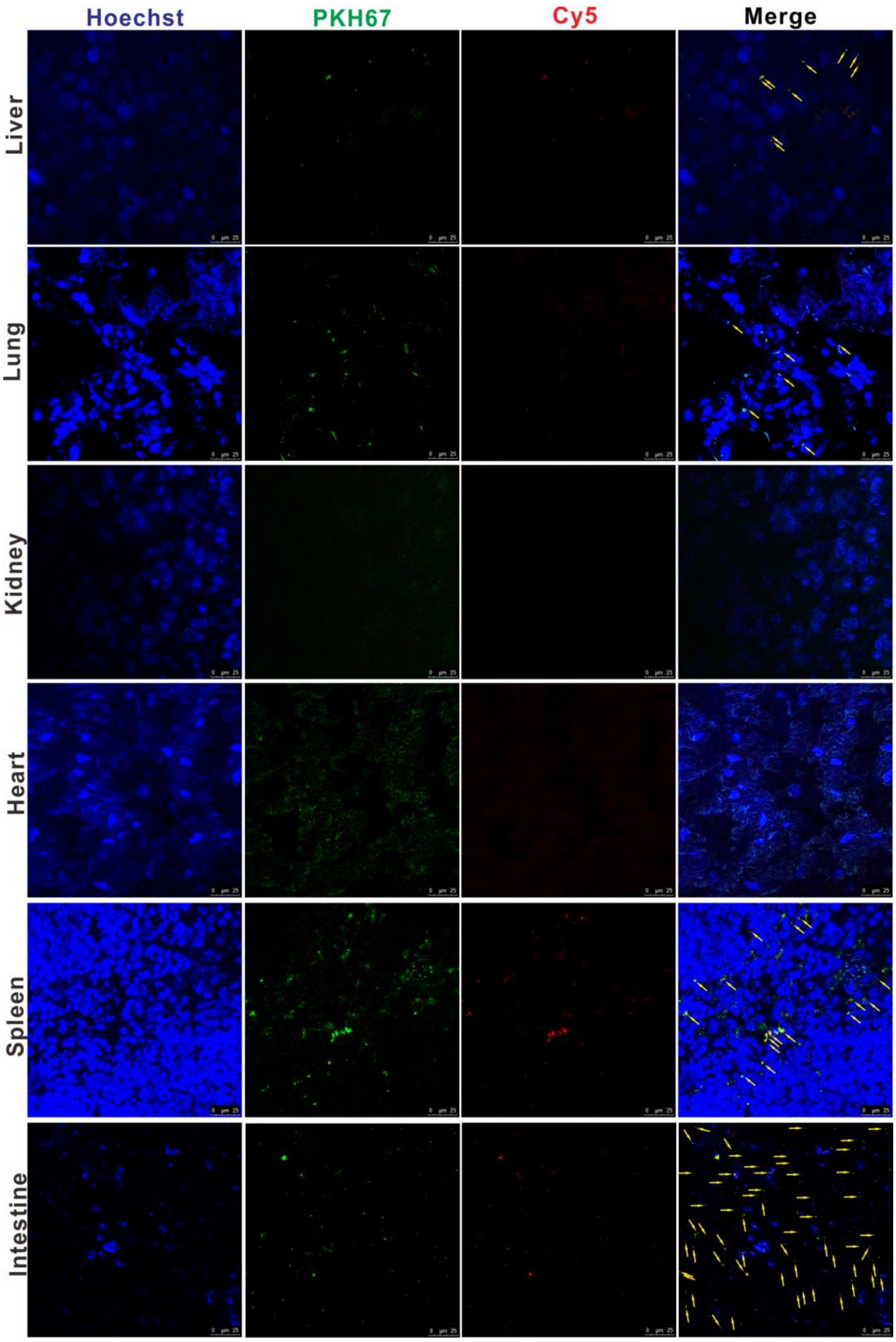

Supplementary Fig. 29. Distribution of MSN-Exo in different organs after 4 h injections of A-Exo and MSN-AP. PKH67-labelled A-Exo (10 μg) were injected into mice through tail vein. Ten min later, MSN-AP-Cy (5 mg/kg) were injected. Mice were sacrificed after 4 hours and then kidney, small intestine and lung were frozen-sectioned, and the tissue distribution of MSN-Exo (yellow) was analyzed by confocal microscopy. Source data are provided as a Source Data file.

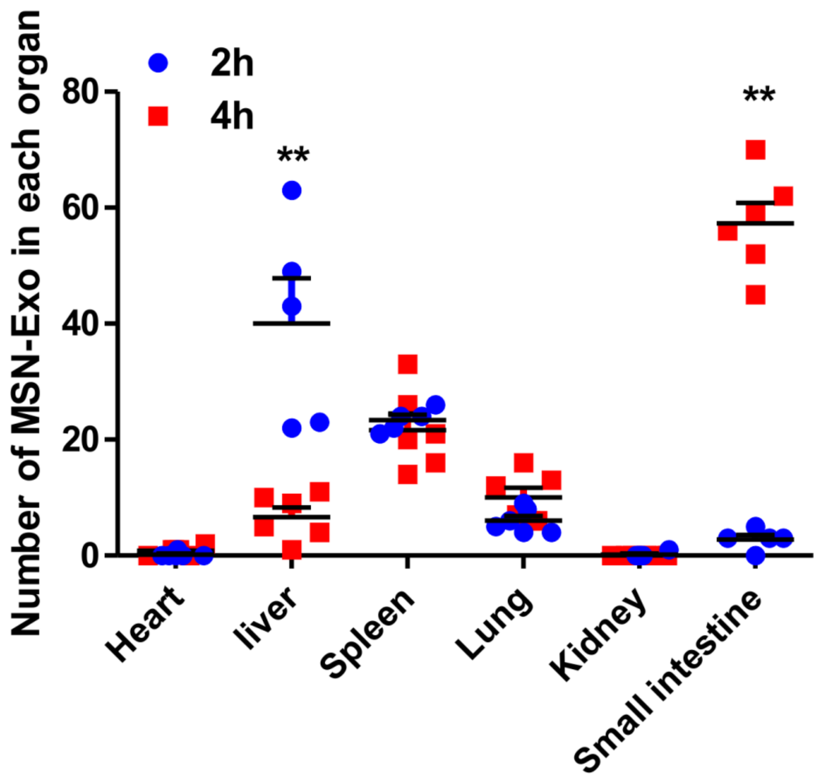

Supplementary Fig. 30. Quantification of the distribution of MSN-Exo in different organs after 2 h and 4 h injection. The Y-axis represents the average number of the 5-6 randomly–selected single fields of visions. Bars represent mean ± s.e.m. **, *P*< 0.01; unpaired two-tailed *t*-test. Source data are provided as a Source Data file.

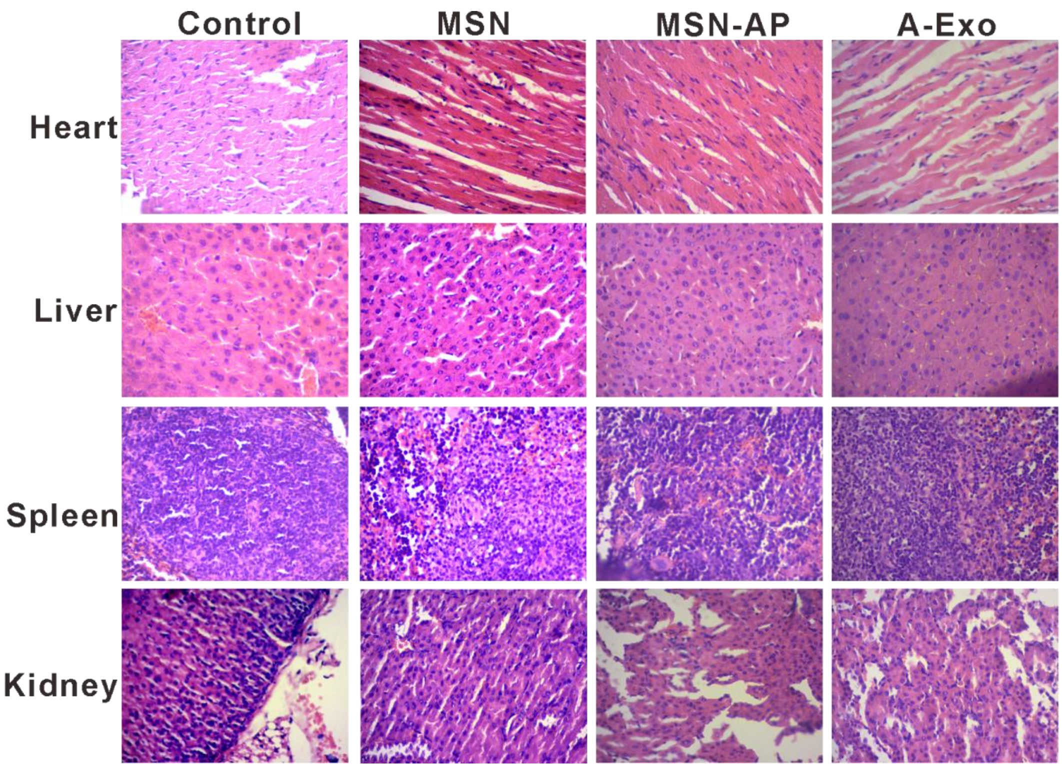

Supplementary Fig. 31. H&E staining of various mouse organs. After treatments with saline, MSN, MSN-AP and A-Exo, the mouse organs were H&E-stained, and no pathological changes were found. Source data are provided as a Source Data file.

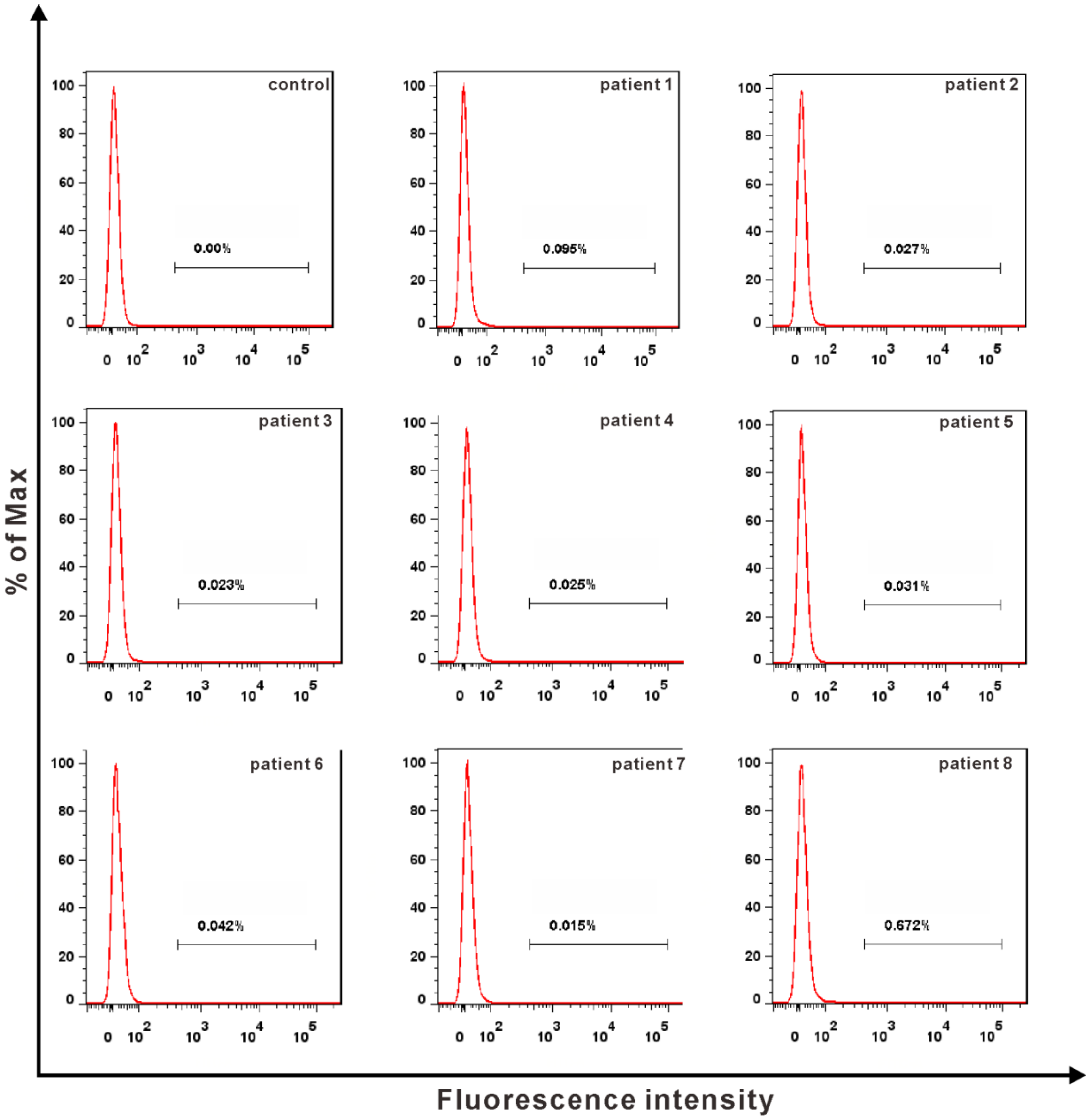

Supplementary Fig. 32. Flow cytometry analysis of patient's EGFR-exosomes captured by MSN-AP. Flow cytometry analysis of ability of MSN-AP to capture EGFR-exosomes derived from lung cancer patients' blood samples. Control: beads incubated with MSN-AP-Cy only. Source data are provided as a Source Data file.
