# Supplementary material for: Eliminating blood oncogenic exosomes into the small intestine with aptamer-functionalized nanoparticles
Source: Nat Commun. 2019 Dec 2;10:5476. doi: 10.1038/s41467-019-13316-w (PMC6889386; doi:10.1038/s41467-019-13316-w)
Supplement: Supplementary file 3 — Reporting Summary [file 41467_2019_13316_MOESM3_ESM.pdf]

nature research

Corresponding author(s): Lee Jia

Last updated by author(s): Jul 25, 2019

Reporting Summary

Nature Research wishes to improve the reproducibility of the work that we publish. This form provides structure for consistency and transparency in reporting. For further information on Nature Research policies, see Authors & Referees and the Editorial Policy Checklist.

Statistics

For all statistical analyses, confirm that the following items are present in the figure legend, table legend, main text, or Methods section.

n/a	Confirmed	
☐	☒	The exact sample size (*n*) for each experimental group/condition, given as a discrete number and unit of measurement
☐	☒	A statement on whether measurements were taken from distinct samples or whether the same sample was measured repeatedly
☐	☒	The statistical test(s) used AND whether they are one- or two-sided<br>*Only common tests should be described solely by name; describe more complex techniques in the Methods section.*
☒	☐	A description of all covariates tested
☐	☒	A description of any assumptions or corrections, such as tests of normality and adjustment for multiple comparisons
☐	☒	A full description of the statistical parameters including central tendency (e.g. means) or other basic estimates (e.g. regression coefficient) AND variation (e.g. standard deviation) or associated estimates of uncertainty (e.g. confidence intervals)
☒	☐	For null hypothesis testing, the test statistic (e.g. *F*, *t*, *r*) with confidence intervals, effect sizes, degrees of freedom and *P* value noted<br>*Give P values as exact values whenever suitable.*
☒	☐	For Bayesian analysis, information on the choice of priors and Markov chain Monte Carlo settings
☒	☐	For hierarchical and complex designs, identification of the appropriate level for tests and full reporting of outcomes
☒	☐	Estimates of effect sizes (e.g. Cohen's *d*, Pearson's *r*), indicating how they were calculated

Our web collection on statistics for biologists contains articles on many of the points above.

Software and code

Policy information about availability of computer code

Data collection	BioRadCFXManager3.0, Image Lab, Leica Microsystems CMS GmbH, NanoScopeAnalysis, Flow Jo 7.6.1, NanoApplication and office 2010 were used to collect data.
Data analysis	GraphPad 5.0 and SPSS statistics 17 were used to analyze data.

For manuscripts utilizing custom algorithms or software that are central to the research but not yet described in published literature, software must be made available to editors/reviewers. We strongly encourage code deposition in a community repository (e.g. GitHub). See the Nature Research guidelines for submitting code & software for further information.

Data

Policy information about availability of data

All manuscripts must include a data availability statement. This statement should provide the following information, where applicable:

- Accession codes, unique identifiers, or web links for publicly available datasets
- A list of figures that have associated raw data
- A description of any restrictions on data availability

All source data, associated code and additional results of this study are available from the corresponding author upon reasonable request.

Field-specific reporting

Please select the one below that is the best fit for your research. If you are not sure, read the appropriate sections before making your selection.

☒ Life sciences ☐ Behavioural & social sciences ☐ Ecological, evolutionary & environmental sciences

Life sciences study design

All studies must disclose on these points even when the disclosure is negative.

Sample size	The sample size was determined by related reports and similar experiments.
Data exclusions	No data was excluded from the analysis
Replication	All attempts in replication were successful.
Randomization	All the groups in this study are random.
Blinding	The investigators were blinded to group allocation during data collection

Reporting for specific materials, systems and methods

We require information from authors about some types of materials, experimental systems and methods used in many studies. Here, indicate whether each material, system or method listed is relevant to your study. If you are not sure if a list item applies to your research, read the appropriate section before selecting a response.

Materials & experimental systems

n/a	Involved in the study	
☐	☒	Antibodies
☐	☒	Eukaryotic cell lines
☒	☐	Palaeontology
☐	☒	Animals and other organisms
☒	☐	Human research participants
☒	☐	Clinical data

Methods

n/a	Involved in the study	
☒	☐	ChIP-seq
☐	☒	Flow cytometry
☒	☐	MRI-based neuroimaging

Antibodies

Antibodies used

anti-CD9 antibody (abcam, EPR2949, ab92726), anti-CD63 antibody (abcam, C-terminal, ab230414), anti-EGFR antibody (abcam, EP38Y, ab52894);F4/80(1:50, Abcam, FITC, AB60343), EpCAM(1:100, invitrogen, FITC, 11-5791-82)

Validation

anti-CD9 antibody (abcam, EPR2949, ab92726),
description
Rabbit monoclonal [EPR2949] to CD9
Host species
Rabbit
Tested applications
Suitable for: WB, IP, IHC-Pmore details
Species reactivity
Reacts with: Mouse, Rat, Human
anti-CD63 antibody (abcam, C-terminal, ab230414),:
Description:
Mouse monoclonal [TS63] to CD63
Application:
Flow Cyt, ICC/IF, IHC-P, WB
Reactivity:
Human
anti-EGFR antibody (abcam, EP38Y, ab52894)
Description:
Rabbit monoclonal [EP38Y] to EGFR
Application:
Flow Cyt, ICC/IF, IHC-Fr, IHC-P, IP, WB
Reactivity:
Mouse, Rat, Human
F4/80(1:50, Abcam, FITC, AB60343)
Description:
Rat monoclonal [BM8] to F4/80 (FITC)
Application:
Flow Cyt, ICC/IF, IHC-Fr, IHC-P
Reactivity:
Mouse

Conjugate:
FITC
EpCAM(1:100, invitrogen, FITC, 11-5791-82)
Species Reactivity
Mouse
Published species
Human, Mouse
Host / Isotype
Rat / IgG2a, kappaPublished Applications
Flow Cytometry (Flow)
See 20 publications below
Immunofluorescence (IF)
See 10 publications below
Affinity Purification (AP)
See 1 publication below
Immunohistochemistry (IHC)
See 1 publication below
Immunohistochemistry (Frozen) (IHC (F))

Eukaryotic cell lines

Policy information about cell lines

Cell line source(s)	A549, HELF, LO2 were purchased from the Cell Bank of Type Culture Collection of Chinese Academy of Sciences (Shanghai, China) and HIBEpiC cells were obtained from Shanghai Bioleaf Biotech Co., Ltd. (Shanghai, China). Kupffer cells were isolated as described previously (Gopalakrishnan, S. & Harris, E. N. In vivo liver endocytosis followed by purification of liver cells by liver perfusion. J Vis Exp, (2011))Human umbilical vein endothelial cells (HUVECs) were prepared and maintained as previously described(Lu, Y. et al. Nitric oxide inhibits hetero-adhesion of cancer cells to endothelial cells: restraining circulating tumor cells from initiating metastatic cascade. Sci Rep 4, 4344, (2014).)
Authentication	All the cells in this study were obtained from standard commercial sources authentication or isolated according to reported researches. And the specific proteins were detected.
Mycoplasma contamination	Cells tested negative for mycoplasma
Commonly misidentified lines (See ICLAC register)	None

Animals and other organisms

Policy information about studies involving animals; ARRIVE guidelines recommended for reporting animal research

Laboratory animals	SD rat :female, 6-8 week C57BL/6:femal, 3-5 week Nude mouse: Balb/c, female 3-5week
Wild animals	This study did not involve wild animals.
Field-collected samples	This study did not involve samples collected from the field.
Ethics oversight	Institutional Animal Care and Use Committee (IACUC) of Fuzhou University

Note that full information on the approval of the study protocol must also be provided in the manuscript.

Flow Cytometry

Plots

Confirm that:

☒ The axis labels state the marker and fluorochrome used (e.g. CD4-FITC).

☒ The axis scales are clearly visible. Include numbers along axes only for bottom left plot of group (a 'group' is an analysis of identical markers).

☒ All plots are contour plots with outliers or pseudocolor plots.

☒ A numerical value for number of cells or percentage (with statistics) is provided.

Methodology

Sample preparation	Exosomes were attached to 2.7-µm aldehyde/sulphate latex beads (Invitrogen) by mixing 10 µg of exosomes with 10 µl of beads for 15 min at room temperature with continuous rotation. This suspension was diluted to 1 ml with PBS and rotated for another 30 min. The reaction was stopped with 100 mM glycine and 2% BSA in PBS for 30 min rotation. The exosome-bound beads were washed with 2% BSA in PBS and centrifuged at 15,000 g for 1 min. The beads were blocked with 10% BSA for 30 min with rotation and washed again in 2% BSA and centrifuged for 1 min at 15,000g, and incubated with MSN-AP-Cy- or MSN-AP-Cy (100

	µg/mL) for 30 min rotation at 4°C. The beads were centrifuged at 15,000 g for 1 min and the supernatant was discarded. The beads were washed in 2% BSA and centrifuged at 15,000 g for 1 min. The blank beads alone were used as a control.
Instrument	BD FACSAria III flow cytometry
Software	FlowJo 7.6.1
Cell population abundance	The beads incubated with secondary antibody alone were used as control, and the preliminary FSC/SSC gates were established according to the characteristic population of control. The exosome-beads conjugation abundance were obtained by the gate of control group.
Gating strategy	The beads incubated with secondary antibody alone were used as control, and the preliminary FSC/SSC gates were established according to the characteristic population of control. The control group as negative population, the fluorescence exceeded the control group as positive population.

☒ Tick this box to confirm that a figure exemplifying the gating strategy is provided in the Supplementary Information.
